# Axon topography of layer 6 spiny cells to orientation map in the primary visual cortex of the cat (area 18)

**DOI:** 10.1007/s00429-016-1284-z

**Published:** 2016-08-18

**Authors:** Fuyuki Karube, Katalin Sári, Zoltán F. Kisvárday

**Affiliations:** 1 Laboratory for Cortical Systems Neuroscience, Department of Anatomy, Histology and Embryology, University of Debrecen, Debrecen, 4032 Hungary; 2 Graduate School of Brain Science, Doshisha University, Tataramiyakodani 1-3, Kyotanabe, Kyoto 610-0394 Japan; 3 Department of Neurosciences Fondamentales, Centre Médical Universitaire, Rue Michel-Servet 1, 4, 1211 Geneva, Switzerland

**Keywords:** Apical dendrite, Bouton overlap, Optical imaging, Pyramidal cell, 3D reconstruction

## Abstract

To uncover the functional topography of layer 6 neurons, optical imaging was combined with three-dimensional neuronal reconstruction. Apical dendrite morphology of 23 neurons revealed three distinct types. Type Aa possessed a short apical dendrite with many oblique branches, Type Ab was characterized by a short and less branched apical dendrite, whereas Type B had a long apical dendrite with tufts in layer 2. Each type had a similar number of boutons, yet their spatial distribution differed from each other in both radial and horizontal extent. Boutons of Type Aa and Ab were almost restricted to the column of the parent soma with a laminar preference to layer 4 and 5/6, respectively. Only Type B contributed to long horizontal connections (up to 1.5 mm) mostly in deep layers. For all types, bouton distribution on orientation map showed an almost equal occurrence at iso- (52.6 ± 18.8 %) and non-iso-orientation (oblique, 27.7 ± 14.9 % and cross-orientation 19.7 ± 10.9 %) sites. Spatial convergence of axons of nearby layer 6 spiny neurons depended on soma separation of the parent cells, but only weakly on orientation preference, contrary to orientation dependence of converging axons of layer 4 spiny cells. The results show that layer 6 connections have only a weak dependence on orientation preference compared with those of layers 2/3 (Buzás et al., J Comp Neurol 499:861–881, 2006) and 4 (Karube and Kisvárday, Cereb Cortex 21:1443–1458, 2011).

**Electronic supplementary material:**

The online version of this article (doi:10.1007/s00429-016-1284-z) contains supplementary material, which is available to authorized users.

## Introduction

Unraveling the connectivity of individual cortical neurons is an important step towards understanding their placement and role in the cerebral cortex. One of the approaches that has been used successfully is to compare connectivity features of single cortical neurons with functional architectures and determine their interrelationship, whereby single cell connectivity can be directly related to the spatial distribution of functional properties. Since the neocortex is a six-layered structure and neurons show marked structural and functional differences depending on laminar location, the above-mentioned comparison must be made at best for each layer separately. For the primary visual cortex, it is known that in the upper layers long-range horizontal connections link regions which represent similar functional attributes such as orientation preference (in cat: Gilbert and Wiesel 1989; Kisvárday et al. 1997; but see also Martin et al. 2014, in monkey: Malach et al. 1993; Yoshioka et al. 1996; in tree shrew: Bosking et al. 1997). When, however, deeper cortical layers are under scrutiny, a marked change in the morphological and functional interrelationship is observed compared to their upper layer counterparts (Hubel and Wiesel 1962; Gilbert 1977; Tsumoto et al. 1978; Henry et al. 1979; Harvey 1980a, b; Martin and Whitteridge 1984). Indeed, it was shown that the orientation distribution of layer 4 (L4) cortico-cortical connections differs from that of the superficial layers, i.e., the connectivity distribution of single L4 spiny neurons is not only less iso-orientation specific (Yousef et al. 1999), but also functional selectivity varies from axon branch to axon branch for the same L4 spiny neuron (Karube and Kisvárday 2011).

Layer 6 (L6) represents one of the chief output layers of the visual cortex to other cortical areas and subcortical structures (Briggs 2010; Thomson 2010). On the other hand, it receives inputs from the dorsal lateral geniculate nucleus (LGN; LeVay and Gilbert 1976; da Costa and Martin 2009; Bannister et al. 2002) and the visual claustrum (LeVay and Sherk 1981; da Costa et al. 2010) and thus establishes cortico-subcortical loops (Gilbert and Kelly 1975; LeVay and Sherk 1981; McCourt et al. 1986; Katz 1987). As a consequence, the connections of L6 spiny cells must have an impact on the entire visual signaling pathway, including other cortical areas as well as subcortical visual centers (for review, see Cudeiro and Sillito 2006; Sillito et al. 2006; Briggs 2010; Thomson 2010). In line with this, there are studies which indicate that L6 neurons are functionally heterogeneous in cat (Hirsch et al. 1998) and other species (Usrey and Fitzpatrick 1996; Wiser and Callaway 1996). For example, in rodent, L6 neurons are assumed to be involved in gain control of visually evoked responses both in cortex and thalamus (Olsen et al. 2012; Bortone et al. 2014) and results of a more recent study indicates that the functional tuning properties of cortically projecting and those of thalamic projecting L6 neurons differ markedly from each other (Velez-Fort et al. 2014). Although a newly developed method allows direct imaging of deep layers in rodents (Andermann et al. 2013; Masamizu et al. 2014), preserving the integrity of anatomical connection of the imaged regions in not resolved.

L6 is also named as multiform layer composed of a heterogeneous neuron population in cat (Gilbert 1977; Harvey 1980b; Katz 1987; Grieve and Sillito 1991a, b, 1995; Hirsch et al. 1998), tree-shrew (Usrey and Fitzpatrick 1996), monkey (Lund and Boothe 1975; Wiser and Callaway 1996) and rodents cortices (Zhang and Deschenes 1997; Zarrinpar and Callaway 2006; Kumar and Ohana 2008; Watakabe et al. 2012). In the cat primary visual cortex, L6 pyramidal neurons are commonly distinguished on the basis of apical dendrite morphology (Lund and Boothe 1975; Katz 1987), which correlates with their subcortical projection patterns. Accordingly, L6 pyramidal cells have been considered to form three main populations: (1) ~50 % of L6 cells project into the LGN (Gilbert and Kelly 1975; McCourt et al. 1986; Katz 1987; Budd 2004) and have a short apical dendrite typically terminating in L4 (type 1 and 2 cells in Katz 1987); (2) <10 % of cells project to the visual claustrum (LeVay and Sherk 1981; Katz 1987) and characterized by a long, slender apical dendrite towards superficial layers; and (3) the remaining cell population provides cortico-cortical connections (McCourt et al. 1986; Katz 1987) either locally, i.e., intrinsic cortico-cortical connections, or to other cortical regions, i.e., extrinsic cortico-cortical connections. It should be noted that a marked difference between L6 neuron types concerns not only morphology, but also their biochemical content. For example, in the non-human primate, L6 cortico-thalamic neurons lack the activity-related neuromodulator synaptic zinc, whereas a subpopulation of cortico-cortical neurons contains synaptic zinc (Ichinohe et al. 2010, for review, see Rockland 2014).

In this study, our aim is to analyze the functional topography of different types of L6 neurons and determine their axonal bouton distribution patterns in relation to local orientation map obtained with intrinsic signal optical imaging in area 18 of the cat primary visual cortex (Payne and Peters 2002). To investigate the potential difference between different types of neuron, we applied a morphological classification of L6 spiny cells based on a quantitative comparison of apical dendrite morphology (see also Katz 1987). In addition to this, in a few cases the functional topography of nearby L6 pyramidal cells was compared with that of L4 spiny neurons. We will discuss how the L6 circuitry is organized with regard to functional architectures and in the context of other cortical layers.

## Materials and methods

Nine adult cats (1.8–3.4 kg) were used in this study. All experiments were performed according to institutional (Local Committee at the University of Debrecen) and governmental requirements [Hungarian Enactment of the Protection of Animals (1998.XXVIII.) Section 4] and the guidelines of the European Convention for the Protection of Vertebrate Animals Used for Experimental and Other Scientific Purposes (Strasbourg, 18.III, 1986). The detailed method used here was previously reported (Karube and Kisvárday 2011).

### Animal surgery

Anesthesia was induced by intra-muscular injection of a mixture of ketamine (7 mg/kg of Ketavit, Pharmacia and Upjohn GmbH, Erlangen, Germany) and xylazine (1 mg/kg of Primazin, Alfasan, Woerden, The Netherlands). Eye drops of Isopto-Max (Alcon Pharma GmbH, Freiburg, Germany) containing antibiotics for protecting the corneal surface, 5 % phenylephrine-hydrochloride (Neosynephrin-POS, Ursapharm, Saarbrücken, Germany) for relaxing the nictitating membrane and 1 % atropine sulfate (Atropine-POS, Ursapharm) for dilating the pupils were applied. All surgical wounds and pressure points were treated with the topical anesthetic, Lidocaine (Xylocaine gel, Astra Chemicals, Wedel/Holstein, Germany). A catheter was inserted in the femoral artery and a Y-shaped cannula in the trachea. For muscle relaxation and nutrition, a mixture of alcuronium chloride (0.15 mg/kg/h of Alloferin, ICN Pharmaceuticals Germany GmbH, Frankfurt, Germany) and glucose (24 mg/kg/h) diluted in Ringer solution was infused through the arterial catheter. In some animals, gallamine tetrathiodide was used instead of Alloferin. A few seconds after the initial dose of muscle relaxant (4 ml/kg), ventilation was commenced with a mixture of N_2_O/O_2_ (70 %/30 %) gas containing 0.3–0.6 % halothane using an Ugo-Basile cat/rabbit ventilator (10–13 strokes/min, 50–70 ml per stroke). Blood pressure (100–135 mmHg), end-tidal CO_2_ (3.8 ± 0.3 %), body temperature (~38.5 °C) were continuously monitored throughout the experiments. Craniotomy was made in the posterior part of both hemispheres to expose area 18 of the visual cortex [Horsely–Clark coordinates antero-posterior (AP): (−5)-(+15), latero-medial (LM): (+0.5)–(+3.5)] (Tusa et al. 1979). A metal chamber (diameter, 30 mm) was fixed to the skull using dental cement (Paladur, Heraeus Kulzer, Wehrheim, Germany). The eyes were fitted with appropriate correction lenses to focus on the monitor screen (GDM-F520, Sony Electronics Inc., Park Ridge, NJ, USA). Viewing distance was 28.5 or 57 cm. During the experiments, eye positions were repeatedly checked on the basis of tapetal back projection of retinal blood vessels.

### Optical imaging of intrinsic signals

Optical imaging was carried out to map orientation columns in the central visual representation of area 18 (Horsley–Clarke coordinates AP, 0–12 and LM, 0.5–3.5). Dura mater was removed and the chamber filled with silicon oil (50 cSt viscosity; Aldrich, Milwaukee, WI, USA). The vascular pattern of the cortical surface was imaged using green light (*λ* = 545 ± 10 nm). During imaging of intrinsic signals, the cortex was illuminated with orange light (*λ* = 609 ± 5 nm) using fiber-optic cables fitted with either an adjustable fiber-optic head-lens or a fiber-optic ring fixed to the objective. Intrinsic signals were recorded with a CCD camera (CS8310C, Tokyo Electric Industry Inc., Tokyo, Japan) in slow-scan mode through two objectives arranged in a ‘tandem configuration’ (SMC Pentax A 1:1.25, 50 mm). During data acquisition, the camera was focused 700 μm below the cortical surface. Images were acquired by a computer using the Imager 2001 and VDAQ software (Optical Imaging Inc., New York, NY, USA). Visual stimuli were generated using the VSG Series Three (Cambridge Research System, Oxford, UK) system. Stimuli were presented on a B/W monitor (SONY, GDM-F520, 100 HZ, non-interlaced mode) placed at 28.5 cm in front of the cat’s eye. They contained full field square wave gratings with high contrast at eight equally spaced orientations that moved forth and back (bi-directional drift) along the orthogonal axis of the orientation. The mean luminescence of the stimuli including the blank was 13 lx. Spatial and temporal frequency was optimized for area 18 (Movshon et al. 1978; Orban 1984) and each stimulus was presented 25–56 times in a pseudo-random manner. Either the blank screen or a stationary image of the next stimulus was presented during interstimulus interval lasting for 10 s. Image acquisition commenced 1 s after stimulus onset and lasted for 4500 ms during which ten data frames were collected. The camera frames underwent spatial summation (two times binning) resulting in a spatial resolution of 21.28 μm per image pixel. Typically, gratings <0.2 cycle per degree and >1 Hz temporal frequency yielded strong activity change and elongated orientation domains characteristic for area 18 (Swindale et al. 1987; Bonhoeffer and Grinvald 1993; Bonhoeffer et al. 1995; Ohki et al. 2000; Shmuel and Grinvald 2000).

### Electrophysiological recordings

For confirming optical imaging results, single- and multi-unit responses to visual stimulation were recorded extracellularly to define the minimum spatial response field (RF), preferred orientation and direction of motion and ocularity using hand-held stimulation at random locations within the mapped area. To this end, glass electrodes (8–10 μm of tip diameter) were filled with 0.5–1 M NaCl (~5 MΩ). The pipettes were advanced perpendicularly to the cortical surface using a manual micro-drive fitted with a digital depth meter (10 μm accuracy, Sylvac SA, Crissier, Switzerland). Along each electrode track single and multi-units were recorded at several depths (300–2000 µm) from the cortical surface. To stabilize recordings, after advancing the electrode into the cortex, the chamber was filled with 3 % agar and covered with low-melting point paraffin (~43 °C, Merck, Darmstadt, Germany). Signals were amplified by AxoClamp-2A (Axon Instruments, Foster City, Canada) and filtered (0.3–10 kHz). Spike activity was monitored using a window discriminator (World Precision Instruments Inc., Sarasota, FL, USA) coupled to an audio monitor. The recorded units had large minimum discharge field (average: 9.5° ± 6.8° of diagonal length, *N* = 71 from six animals). In some cases RF had large size (maximum, 51°), especially at depths corresponding to layers 5/6.

### Tracer injection, histology, and neuron reconstruction

At selected locations in the optically imaged regions, small extracellular tracer injections were carried out in layer 6 (>1200 μm below the cortical surface). Care was taken in choosing locations which represented flat parts of the lateral gyrus so that an interpretation of the labeled structures with respect to the columnar organization of orientation preference could be readily addressed. For example, cortical locations 1 mm from the inter-hemispheric midline, near to the lateral sulcus and major blood vessels were avoided (see Supplementary Figure 1). In each animal several such injections were deposited (6, 10, 11, 10, 8 and 12 injections, respectively) with a lateral separation of 0.5–1 mm. For neuronal labeling, 1–2 % biotinylated dextran amine (BDA, 10000 MW, Thermo Fisher Scientific, Waltham, MA) was diluted in 0.5–1 M NaCl and filled in recording glass micropipettes (8–10 μm of tip diameter). After unit recordings (see above), BDA was injected thorough the same glass micropipette. In this way, we could visualize the location and approximate depth of recordings. For each injection, positive 0.8 µA square wave current was applied for 20 min (250 ms ON/250 ms OFF). In order to help aligning the histological sections with optical images, 5–8 reference penetrations were placed in the region of interest using empty glass pipettes (~20 µm tip diameter) at stereotaxically determined locations (Supplementary Fig. 1; for details, see also Buzás et al. 1998; Karube and Kisvárday 2011). Finally, the animals received a lethal dose of anesthetics and were perfused transcardially with Tyrode’s solution (~3 min) followed by 2 L of fixative containing 2 % paraformaldehyde (PF, Merck) and 0.1 % gultaraldehyde (GA, Merck) in 0.1 M phosphate buffer (PB, pH 7.4). Tissue blocks containing the region of interest were dissected and 80-μm-thick sections cut on a vibratome (Leica S-1000, Wetzlar, Germany). Importantly, the sectioning plane was set as parallel as possible with the imaged cortical surface. Washing of sections was carried out at room temperature (RT), whereas all other steps of the histological procedure at 4 °C. Briefly, sections were washed in PB (10 min) and 0.05 M Tris buffered saline (TBS, 2× 10 min) followed by incubation in avidin–biotin-complexed HRP [ABC-Elite (Vector, Burlingame, CA, USA) at 1:200 dilution in 0.05 M TBS containing 0.1 % Triton X] overnight. After washing in TBS (10 min) and tris-buffer (TB, pH 7.6), the sections were incubated in 0.05 % of 3,3-diaminobenzidine (DAB, Sigma-Aldrich, St. Louis, MO, USA) in TB containing 0.0025–0.005 % CoCl_2_. DAB was visualized in the presence of H_2_O_2_ (0.01 % at final concentration, for 1–3 min). The reaction was stopped by adding an excess of TB in succession. The sections were post-fixed in OsO_4_ (1 % in 0.1 M PB, 45 min, Sigma-Aldrich) and dehydrated in ascending series of ethanol and two times propylene oxide (Merck). Then the sections were transferred in resin (Durcupan, Sigma-Aldrich) for 24 h, mounted on slides, coverslipped and cured at 56° for 24 h (Somogyi and Freund 1989).

**Fig. 1:**
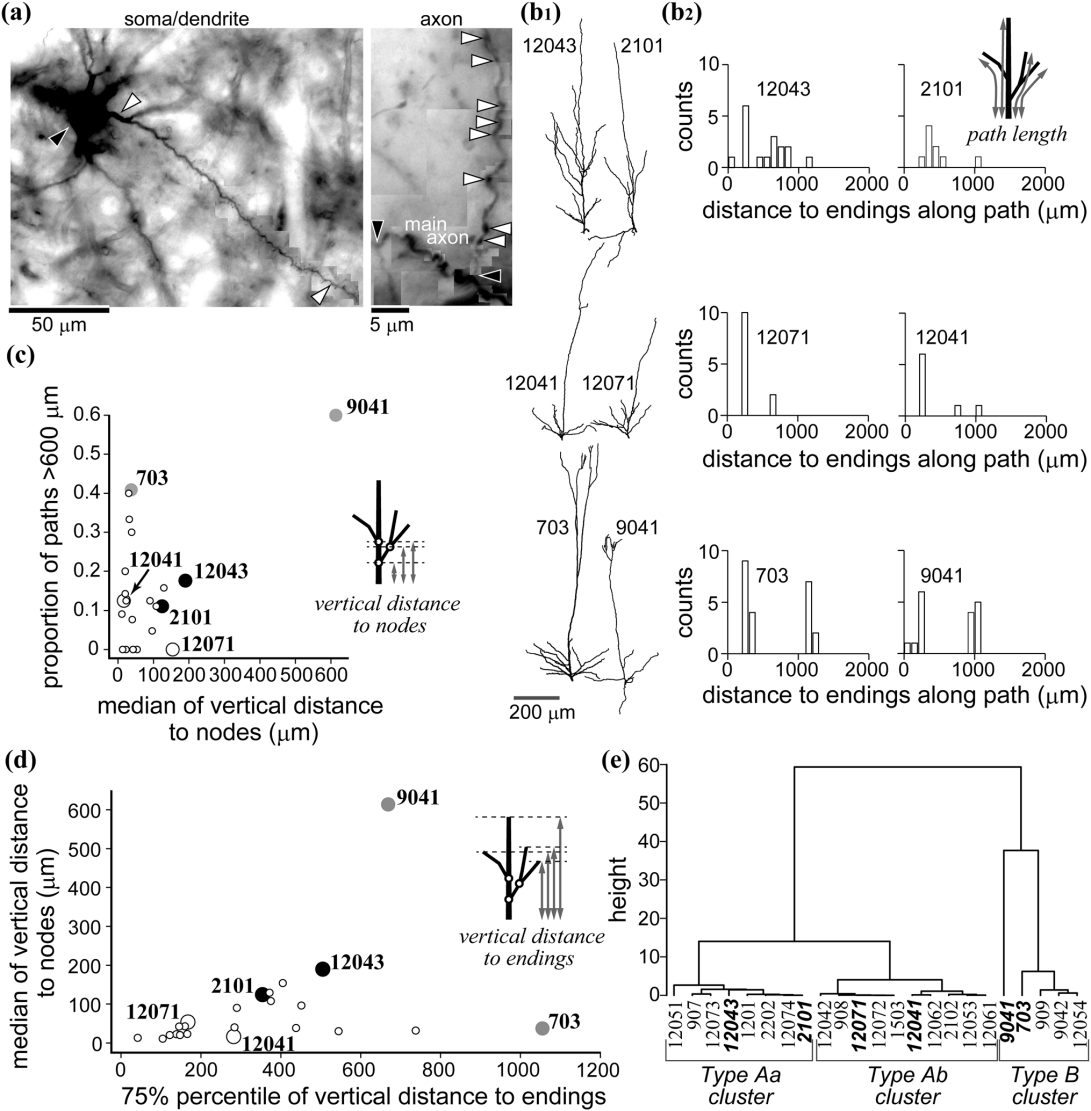
Morphological classification of reconstructed L6 spiny cells. **a** An example of a labeled layer 6 neuron (cell 12074). *Left* Spine-bearing dendrites are issued from the soma (*black arrowhead*). One of the basal dendrites is labeled with *white arrowheads* from base to tip. *Right* Photomontage of a main axon (*black arrowhead*) showing two collaterals and several boutons (*white arrowhead*). **b**_**1**_ Three characteristic apical dendrite types of L6 spiny cells are shown for each type without cell body and basal dendrites. Cell 12043 and 2101 had a short apical dendrite with oblique branches originating from the middle of the main trunk. Cell 12041 and 12071 had a short apical dendrite which issued oblique branches only close to its soma origin. Cell 703 and 9041 had a relatively long apical dendrite with apical tufts, lacking oblique branches around the middle of the main trunk. Apical dendrites were rotated around the pia–white matter axis to represent branches well. **b**_**2**_ Path length frequency distributions representing 3D-distances from the origin of the apical dendrite to each ending of dendritic branches. Path length distributions well reflect differences in apical dendrite morphology shown in (**b**_**1**_). **c** Scatter plot of medians obtained for vertical distance to nodes (ordinate) and 75 % percentile of vertical distances to each ending (abscissa). The cells shown in **b** are numbered. **d** Proportion of long path (>600 μm) values plotted against median of vertical distance to nodes values. Cells with different apical dendrite morphology indicate clustering (c.f. numbered cells and **b**). **e** Dendrogram of cluster analysis (Ward method) using the above three morphological parameters reveals three distinct groups: Type Aa, Type Ab and Type B. Numbers correspond to cell ID

Strongly labeled single cells in layer 6, i.e., dense DAB deposit in the cell body and even at the tip of dendrites, with typical excitatory cell morphology such as pyramidal shaped soma and obvious spiny dendrites were selected (Fig. 1a) and reconstructed at 1000× magnification using a Leitz DMRB microscope (Leica) and the Neurolucida (v.5.4) reconstruction system (MBF Bioscience Inc., Colchester, VT, USA). Adjoining sections were aligned by using cut ends of corresponding labeled neuronal processes, for example, axons which were traced from one section to a neighboring one. For aligning pairs of sections, at least three such processes were used and their corresponding cut ends fitted on the basis of the least-square algorithm (Buzás et al. 1998; Yousef et al. 1999). Then the cell reconstructions were aligned at high precision with corresponding optical images using the landmarks of reference penetrations each of which resulted in a small tissue scar of about 20–80 μm diameter in the sections. The most superficial section containing pia mater and reference penetrations was used for aligning the neuron reconstruction with the optical image. Commonly, a high-precision match could be achieved with a maximum deviation (alignment error) of three image pixels or 64 µm (Buzás et al. 1998). All data were corrected for tissue shrinkage which was determined by taking into account reference penetration intervals measured in vivo (entry points of reference penetrations were marked in the vascular image) as well as after histological processing (entry points of reference penetration were identified in superficial sections). Accordingly, in the data presented here 87.3–93.6 % shrinkage factor was used.

### Data analysis

Optical imaging data were analyzed using the Mix software (Optical Imaging Inc.) and a custom-made program written in IDL (Research Systems Inc., Colorado, CO, USA), and MATLAB (The MathWorks Inc., Natick, MA, USA). Single condition maps (SCMs) were obtained by images recorded to a particular orientation divided by the “cocktail blank” (Bonhoeffer and Grinvald 1996). To reduce low-frequency noise due to uneven illumination and surface blood vessels, SCMs were first high-pass filtered (boxcar filter, 50-pixel kernel size), then low-pass filtered (boxcar filter, 5-pixel kernel size). Orientation maps were calculated from filtered SCMs using the vector summation method followed by interpolation (Blasdel and Salama 1986; Bonhoeffer and Grinvald 1991). Large blood vessels, typically at the edge of the maps, were masked out and not included in data analysis. Quantitative evaluation of the imaged orientation preference was made on the basis of 10 degrees binning of orientation map values. In this way, the results could be directly compared with previously published data.

Morphological parameters of the reconstructed cells (Table 1) were extracted using MATLAB and NeuroExplorer (MBF Bioscience) routines. Cell type classification was made by cluster analysis with Ward’s method (Ward 1963) using three parameters (schematics are shown in Fig. 1b_2_–d) by statistic computing software ‘R’ (https://www.r-project.org). The first parameter, “path length” was defined as the distance from the origin of an apical dendrite to each ending. The proportion of path length values longer than 600 μm was calculated. The second parameter was vertical distance to nodes which is measured from the origin of an apical dendrite to each node (branch point) parallel to the pia–white matter axis. The median of the values was calculated. The third parameter was vertical distance to branch ends as measured from the origin of an apical dendrite to each dendritic ending. In the latter case, the 75 % percentile of the values was used as the representative. Among the 23 cells of L6, the mean and standard deviation (SD) of each parameter was calculated. Then the mean was extracted from the values of each cell and divided by SD for normalization. Now the mean of the normalized values of 23 cells is zero and the SD equals to one for all three parameters. For the cluster analysis, Euclidian distance among all cells was calculated from the above three parameters and the squared values of the distances were used for Ward method. Apical and basal dendrites morphology was also examined by Sholl analysis (Supplementary Fig. 3).

**Table 1:** Basic morphological parameters of layer 6 pyramidal cells

	Type Aa (*N* = 8)	Type Ab (*N* = 10)	Type B (*N* = 5)	Difference
Apical dendrite				
Length (mm)	4.31 ± 0.95	2.43 ± 0.76	2.82 ± 1.62	Aa > Ab**
Number of nodes	15 ± 4	9 ± 4	11 ± 7	Aa > Ab*
Nodes on the trunk				
>600 μm from the soma	0 ± 1	0 ± 0	4 ± 4	Aa > Ab*
>100 μm from the soma	7 ± 4	2 ± 2	5 ± 5	
Basal dendrite				
Number of trunks	7 ± 4	5 ± 1	7 ± 3	
Total length (mm)	4.16 ± 1.10	5.22 ± 0.93	6.69 ± 3.14	
Total number of nodes	15 ± 5	21 ± 7	21 ± 4	
Axon				
Length (mm)	13.96 ± 6.81	16.00 ± 5.28	20.16 ± 7.18	
Number of nodes	41 ± 22	47 ± 18	44 ± 20	
Number of boutons	1482 ± 765	1308 ± 485	1448 ± 535	
Proportion of superficial layer boutons (%)	4.8 ± 8.9	21.2 ± 19.2	28.7 ± 26.8	
Mean bouton density/100 μm	11.3 ± 3.6	8.6 ± 2.0	8.1 ± 2.4	
Cell body area (μm^2^)	247.78 ± 31.75	264.91 ± 67.7	302.78 ± 230.59	

Values represent mean ± SD. Statistical significance was examined by post hoc Tukey test following ANOVA (* *p* < 0.05; ** *p* < 0.01). Bouton density was shown as the mean of values calculated for each axonal segment separately

Cortical layers were determined on the basis of a host of qualitative features such as relative neuron and nerve fiber density, soma size, and the presence of layer-specific cell types, for example, large pyramidal cells at the layer 3/4 border and in layer 5 (O’Leary 1941; Otsuka and Hassler 1962; Harvey 1980a). It should be noted that due to horizontal sectioning and the lack of counterstaining laminar borders could not always be unequivocally determined across all axon ranges. Hence, for quantitative purposes we subdivided the cortical thickness into superficial (L1–3) and deep layers (L4–6).

Bouton clusters of single cells were identified by partitioning the three-dimensional bouton distributions using a custom-made MATLAB script based on the mean-shift algorithm (Binzegger et al. 2007. For details, see Karube and Kisvárday 2011). In brief, appropriate kernel size for the algorithm was determined by the similarity index (SI; Gusfield 2002; Giurcăneanu and Tăbuş 2004; Binzegger et al. 2007). In the first calculation the kernel size was set to 10 µm, and then increased in 5-µm steps. Then, SI between each consecutive calculation was obtained as 1 − *m*/(*u* − 1), where *m* is the minimum number of points to be eliminated for making two partitions equal, and *u* is the number of total boutons. Then the kernel size (*k*) was specified which provides stable SI (>0.975 for longer than 15 µm range). During calculation, we simply used the distance between the centers of bouton clusters, *k*/2, as threshold to be merged the clusters. The value of 10^−3^ *k* was used for the stop threshold of the mean-shift algorithm. Although our analysis resulted in a relatively larger number of clusters per cell than in the study of Binzegger et al. (2007), our algorithms and the applied calculations were much simpler to use, and basically providing similar results (see Fig. 6, Supplementary Fig. 4 in Karube and Kisvárday 2011).

Angle difference (∆ori) of preferred orientation between parent soma and the boutons was calculated on the basis of their location within the orientation map from which frequency distribution of ∆ori was generated using 10° binning. For comparing the results with previously published findings, ∆ori was additionally binned into three categories representing, respectively, iso (0°–30°), oblique (30°–60°) and cross-orientation (60°–90°) (Kisvárday et al. 1994).

Bouton overlap index (BOI, Figs. 8, 9) was calculated for distal boutons of cell A, which overlapped with distal boutons of cell B as follows:

$${BOI_{A \to B} = \frac{{N_{A\& B} }}{{N_{A} }} \times 1 0 0},$$

where *N*_A&B_ is the number of pixels containing distal boutons of cell A overlapping with distal boutons of cell B, and *N*_A_ is the total number of pixels containing distal boutons of cell A. Distal boutons were defined as those possessed a larger radial separation from the parent soma than the mean radius of the dendritic field (200 μm radius for L4 cells and 250 μm for L6 cells). For BOI, a 3-pixel kernel centered on each bouton of cell A was used in order to search overlapping boutons of cell B (Fig. 8d). It is conceivable that a pair of cells, e.g., cell A and cell B, returned typically two BOI values, since most often *N*_A_ differed from *N*_B_.

### Immunohistochemistry

To determine the exact laminar extent and sublaminar organization of L6 histochemical detection of thalamocortical terminals, vesicular glutamate transporter 2 (VGluT2) was employed (Kaneko et al. 2002; Nahmani and Erisir 2005) on tissue samples taken from the primary visual cortex of three normal adult cats. For this purpose, the fixative contained a mixture of 2 % PF and 0.05 % GA in 0.1 M PB. Tissue block containing areas 17, 18, and 19 was dissected and 20-µm-thick coronal sections cut on vibratome. Sections were immersed in 15 % then 30 % sucrose in PB and freeze-thawed twice in liquid N_2_. In order to reduce non-specific staining (endogenous peroxidase activity), sections were washed with 0.05 M TBS and treated with 1 % H_2_O_2_ in TBS for an hour. Primary antibody to vesicular glutamate transporter 2 (VGluT2, Chemicon International, Temecula, CA) was used (1:5000 in TBS containing 5 % normal goat serum, 2 % bovine serum albumin and 0.5 % Triton X) for 24 h at 4 °C or overnight at RT. After 3 × 10 min rinses in TBS, the sections were incubated with secondary antibody (biotinylated anti-guinea pig antibody (Vector), 1:200) for overnight at 4 °C or 4 h at RT. VGluT2 immunoreactivity was visualized using standard ABC/DAB protocol (see above). The sections were washed in PB, and dry-mounted on a gelatin-coated glass slide, dehydrated and defatted in ascending series of ethanol and xylene and coverslipped in Depex (SERVA Electrophoresis GmbH, Germany).

For double immunohistochemistry, a mixture of anti-VGluT2 and mouse anti-parvalbumin (1:2000, PV235, Swant, Bellinzona Switzerland) was used as primary antibodies. VGluT2 labeling was revealed with biotinylated anti-guinea pig antibody (Vector) using standard ABC/DAB reaction in the presence of CoCl_2_ resulting in dark blue end product. Then, the sections were treated with 2 % NaN_3_ for 10 min followed by 2 % H_2_O_2_ and 0.07 % *p*-cresol in TBS for 15 min to inactivate peroxidase and complete DAB oxidization. For revealing PV labeling, biotinylated anti-mouse IgG antibody (Vector, 1:200) was used as secondary antibody. Here the labeling was developed with ABC/DAB in the absence of CoCl_2_, providing a brown color of the chromogen.

For quantification of VGluT2-positive terminals, light micrographs were taken with an Olympus BX50 (Olympus, Tokyo, Japan) or Leica DMR microscopes equipped with a digital camera (Optronics, Cremona Drive, CA, USA) at 400× magnification. The number of VGluT2-positive terminals in L6 was counted and evaluated using Neurolucida and StereoInvestigator (MBF Bioscience), and Image J (National Institute of Health, USA) software. To better identify laminar and area borders Nissl- and cytochrome oxidase staining (Boyd and Matsubara 1996) of adjacent sections were also used.

Statistical significance among three groups was examined using pairwise Wilcoxon test with Holm’s correction or post hoc Tukey test following ANOVA. For comparison between two groups, Mann–Whitney *U* test was applied. Kolmogorov–Smirnov test was used for comparison of cumulative frequency distribution curves. For categorical data, Fisher’s exact test was used. All data are given as mean ± SD.

## Results

**Fig. 2:**
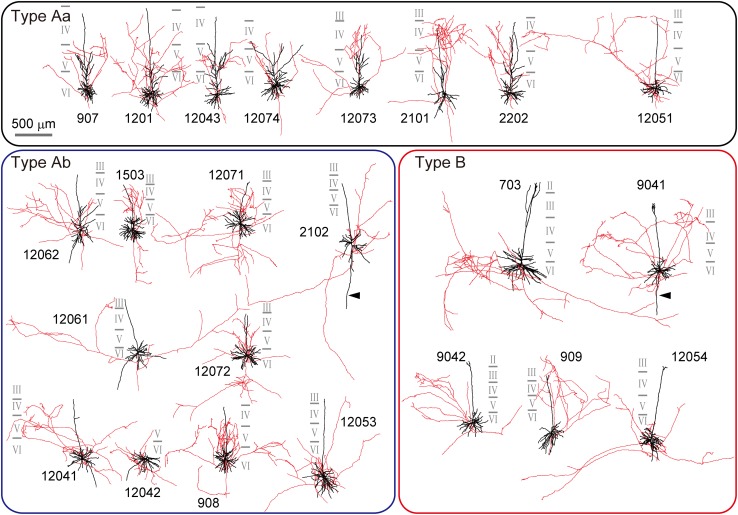
Reconstructions of L6 spiny cells (*N* = 23). Cells are divided into three types, according to the cluster analysis shown in Fig. 1e: Type Aa (*top*), Type Ab (*lower left*) and Type B (*lower right*). See also main text. Reconstructions are rotated around the pia–white matter axis in order to show horizontal extent of their axons. Dendrites and somata are shown in *black*, axons in *red*. *Solid gray lines* close to each cell represent laminar borders which are numbered. *Scale bar* 500 μm

In the present study, 23 L6 spiny cells in the cat primary visual cortex (area 18) were extracellularly labeled (Fig. 1a) and reconstructed in three-dimensions (Fig. 2, Supplementary Fig. 2). Their dendritic and axonal morphology were analyzed comparing with orientation maps obtained by intrinsic optical imaging.

### Morphological types of L6 pyramidal cells

All but one spiny cell in our sample had a distinct apical dendrite and they were identified as pyramidal cells. For the one spiny cell, only short dendrites emerged of which the longest issuing from the pial side of the cell body was considered as apical dendrite (Fig. 2. Nr. 12042). As shown in Figs. 1 and 2, the morphology and spatial extent of apical dendrites varied considerably from cell to cell not only in length, but the distribution of daughter branches (oblique branches and tufts). To quantify these differences three morphological parameters were measured: path length, vertical distance to dendritic endings, and vertical distance to nodes, i.e., dendritic branch points (Fig. 1b_2_–d; see also “Materials and methods”). Examples of path length distribution are shown in Fig. 1b_2_. The histograms well reflect the actual dendrite morphology that depends on the spatial distribution of dendritic branches. Cells with oblique dendritic branches close to the soma had a relatively large proportion of short path values (middle row in Fig. 1b), whereas cells possessing apical dendritic tufts had a relatively large proportion of long path values (bottom in Fig. 1b). Because path length represents three-dimensional distances, it reflects not only laminar distribution of dendritic branches but also informs about their horizontal extent. The two other parameters which were used for quantitative grouping reflected purely the radial extent of apical dendrites. Vertical distance to nodes was defined as the distance parallel to the pia–white matter axis from the origin of the apical dendrite to each dendritic branch point (node) and vertical distance to endings as the vertical distance from the origin of the apical dendrite to each apical dendritic ending (terminal). Scatter plots in Fig. 1c shows the proportion of path length longer than 600 μm against the median of vertical distance to nodes and Fig. 1d the median of vertical distance to nodes against the 75 % percentile of the vertical distance to tips. In both scatter plots different shapes of apical dendrites illustrated in Fig. 1b_1_ were segregated.

By the above three parameters of apical dendrites, cluster analysis assigned our sample of L6 cells (Fig. 2, *N* = 23 cells) into two large clusters referred to as Type A (*N* = 18 cells) and Type B (*n* = 5 cells) (Fig. 1e). Type A cells could be further divided into two subgroups: Type Aa (*N* = 8) and Type Ab (*N* = 10). Type A was composed of cells with a short apical dendrite lacking terminal tufts. Within this group, the apical dendrite of Type Aa typically did not cross the border between L4 and 3 and emitted several oblique branches issuing from the main apical trunk, mainly in L5 and L4. Type Ab also had a short apical dendrite; however, oblique branches emerged only from the proximity of the parent soma. Conversely, Type B possessed a long apical dendrite which entered L2. The proportion of long path values statistically differed between Type Aa and Type B, and Type Ab and Type B (*p* < 0.01), but not between Type Aa and Type Ab. On the other hand, the 75 % percentile of vertical distance from the soma to each apical dendrite terminal was significantly different between all three groups [Type Aa and Ab (*p* < 0.01), Type Aa and B (*p* < 0.01), and Type Ab and B (*p* = 0.02)]. Other morphological parameters of each type are also summarized in Table 1. Most notably, Type Aa had more nodes on proximal part of the apical dendrite (within 100 μm from soma) and a longer total apical dendrite length than Type Ab. As opposed to apical dendrites the spatial extent of basal dendrites did not differ among cell types (Supplementary Fig. 3; but see also Katz 1987). On average 6 ± 2 (range 4–12) basal dendrites emerged from the cell body in all directions. The majority (98 %) of basal dendrites arborized within a 250 μm radius around the soma, although for two cells, a single long basal dendrite emerged towards the white matter (Fig. 2, arrowhead). It should be noted that these cells differed clearly from the so-called inverted pyramidal cells (for review, see Mendizabal-Zubiaga et al. 2007), because they invariably had an apical dendrite towards the pial surface providing a quasi-bipolar character to these cells.

**Fig. 3:**
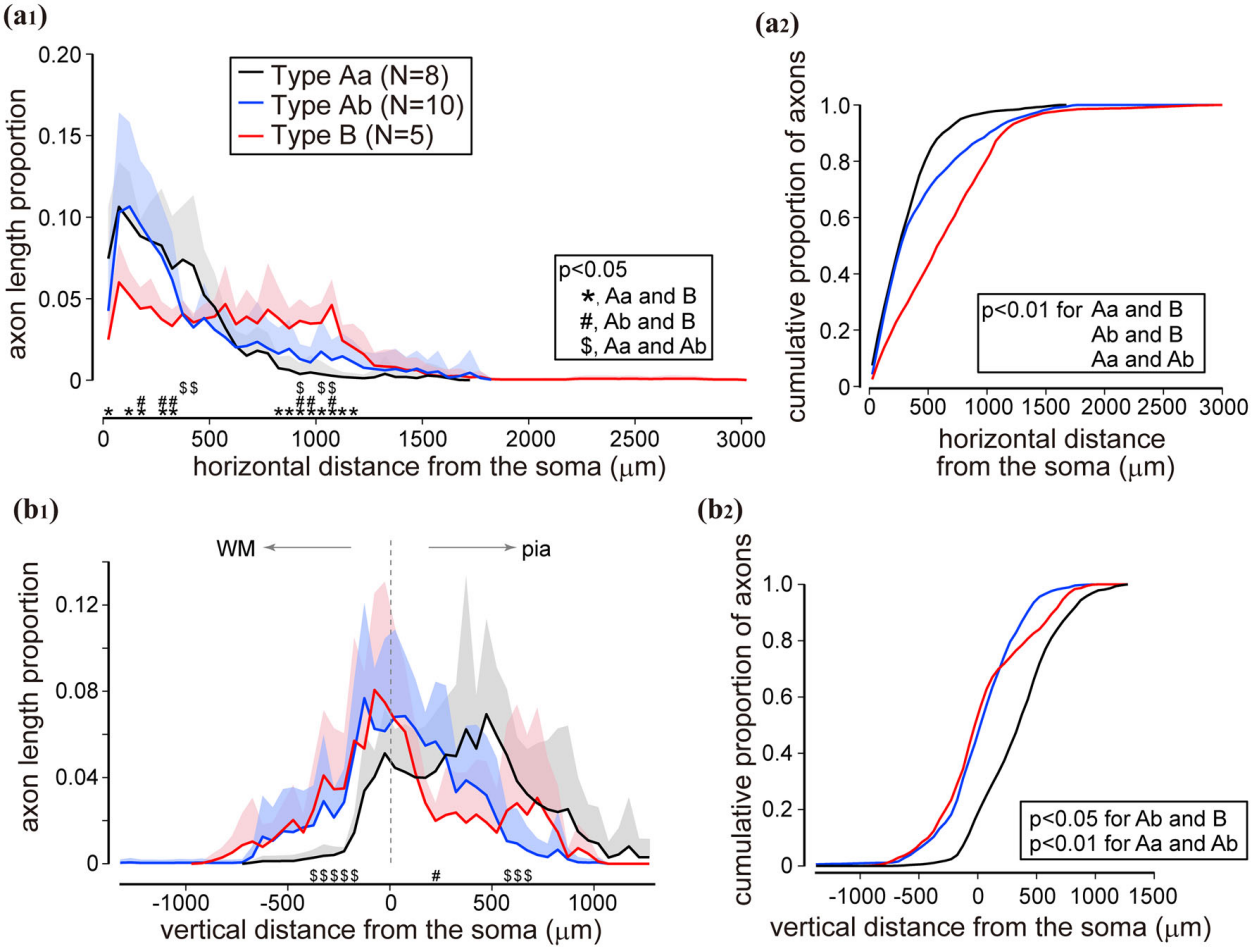
Spatial distribution of axons of the three types of L6 cell. **a**_**1**_ Horizontal distribution of axons. Axon length proportion was plotted against horizontal distance from the soma location. Type Aa (*black line*) and Ab (*blue*) each had sharp peak close to the soma, whereas Type B (*red*) had a relatively uniform distribution up to about 1000 μm. *Shaded area* represent positive SD. Difference between corresponding data points among types was examined using pairwise Wilcoxon test with Holm’s correction. Significant difference (*p* < 0.05) is marked by *asterisk* (*) between Type Aa and B, ^#^between Type Ab and B, and by $ between Type Aa and Ab. **a**_**2**_ Cumulative distribution of data shown in **a**_**1**_. The distribution was significantly different among all types (*p* < 0.01, Kolmogorov–Smirnov test with Bonferroni correction). **b**_**1**_ Vertical distribution of axons. Positive values of distance represent the direction toward the pial surface. Proportion of axon length was also different among cell types. Type Aa had a broad peak around 500 μm from the soma, whereas others did close to the soma. Statistical significance was tested in the same way as in **a**_**1**_. **b**_**2**_ Cumulative distribution of proportion of axons in vertical distance from the soma. Significant difference was observed between Type Ab and B (*p* < 0.05), and Aa and Ab (*p* < 0.01)

We investigated sublaminar distribution of our sample of L6 cells. The mean distance from the top of L6 was 353 ± 181 μm for Type Aa, 230 ± 174 μm for Type Ab and 240 ± 190 μm for Type B. Since the distance values did not differ significantly among cell types (*p* = 0.55 by one-way ANOVA) their distribution can be regarded as unbiased.

### Axon distribution of L6 cell types

Each apical dendritic type could be associated with a characteristic axon distribution as shown in Fig. 3 (for drawings see Fig. 2). With regard to horizontal extent, Type Aa and Ab had a large proportion of axons close to the parent somata, i.e., 82.5 % of axon length for Type Aa and 69.9 % for Type Ab were located within 500 μm around their somata, which, however, decreased rapidly at larger distances (Fig. 3a). Contrary to this, the lateral distribution of axons of Type B contained only less than half (43.1 %) of axon length within 500 μm from the somata. In fact Type B axons had the widest lateral extent beyond 1 mm. Here the differences of horizontal distribution of axons were statistically significant among all three types (pairwise Wilcoxon test in Fig. 3a_1_ and Kolmogorov–Smirnov test in Fig. 3a_2_). Considering the wide lateral extent of the axon and the long apical dendrite of Type B cells, they resembled the so-called “horizontal” L6 cells reviewed by Briggs (2010). On the other hand, the amount of radially distributed axons also differed among types as shown in Fig. 3b. Type Aa had a larger proportion of axons above the cell body level which reached the peak around 500 μm above the soma or corresponding L4 and L5. Below the cell body their proportion decreased rapidly. For the other two types, the overall proportion of axons was the largest in L6, i.e., in the cell body layer and the overlying L5. It should be mentioned that Type B had another smaller peak more superficial depth, around 700–800 μm above the cell body (Fig. 3b_1_). Radial distribution of axons differed between Type Ab and B, and Aa and Ab in cumulative proportion (Kolmogorov–Smirnov test in Fig. 3b_2_). To sum up the above findings, Type Aa axons which had a rather columnar termination field (to some extent Type Ab also) preferred L4 and L5, whereas Type Ab preferred L6. On the other hand, Type B had a considerably wider axonal field extending over 1 mm and was dense in deep layers mainly, but was also present in superficial layers. Important to note that the above quantitative results were consistent with the results of a similar analysis carried out for boutons instead of axon segments (Supplementary Fig. 4).

It is tempting to compare the above morphological properties of both dendrites and axons with earlier reports of L6 spiny cell morphology (Katz 1987; Hirsch et al. 1998). In this regard, our Type Aa neurons can correspond to those projecting into the LGN (cortico-thalamic cells), Type Ab to other cortical areas (cortico-cortical cells), and Type B neurons those projecting into the claustrum (cortico-claustral cells). Although, the possibility remains that as Katz’s Type 2 cells (Katz 1987), all types described here may contain non-projection cells. Nonetheless, multiple projection types of cells cannot be excluded either. Next, the axonal distribution of each type to functional architectures was investigated.

### Bouton clustering of L6 cells

In the mammalian visual cortex, boutons of many superficial layer (L2/3) pyramidal cells are known to terminate in distinct clusters forming patchy projection of the axons (Rockland and Lund 1982; Gilbert and Wiesel 1983; Rockland and Lund 1983; Martin and Whitteridge 1984; Kisvárday and Eysel 1992; Binzegger et al. 2007). L4 neurons also provide clustered projections although with different spatial constraints compared to their superficial layer counterparts (Karube and Kisvárday 2011). However, there is no comparable data for deep layer neurons despite the fact that in a few studies clustered axons provided by L5 and L6 cells have been described (Gabbott et al. 1987; Binzegger et al. 2007).

**Fig. 4:**
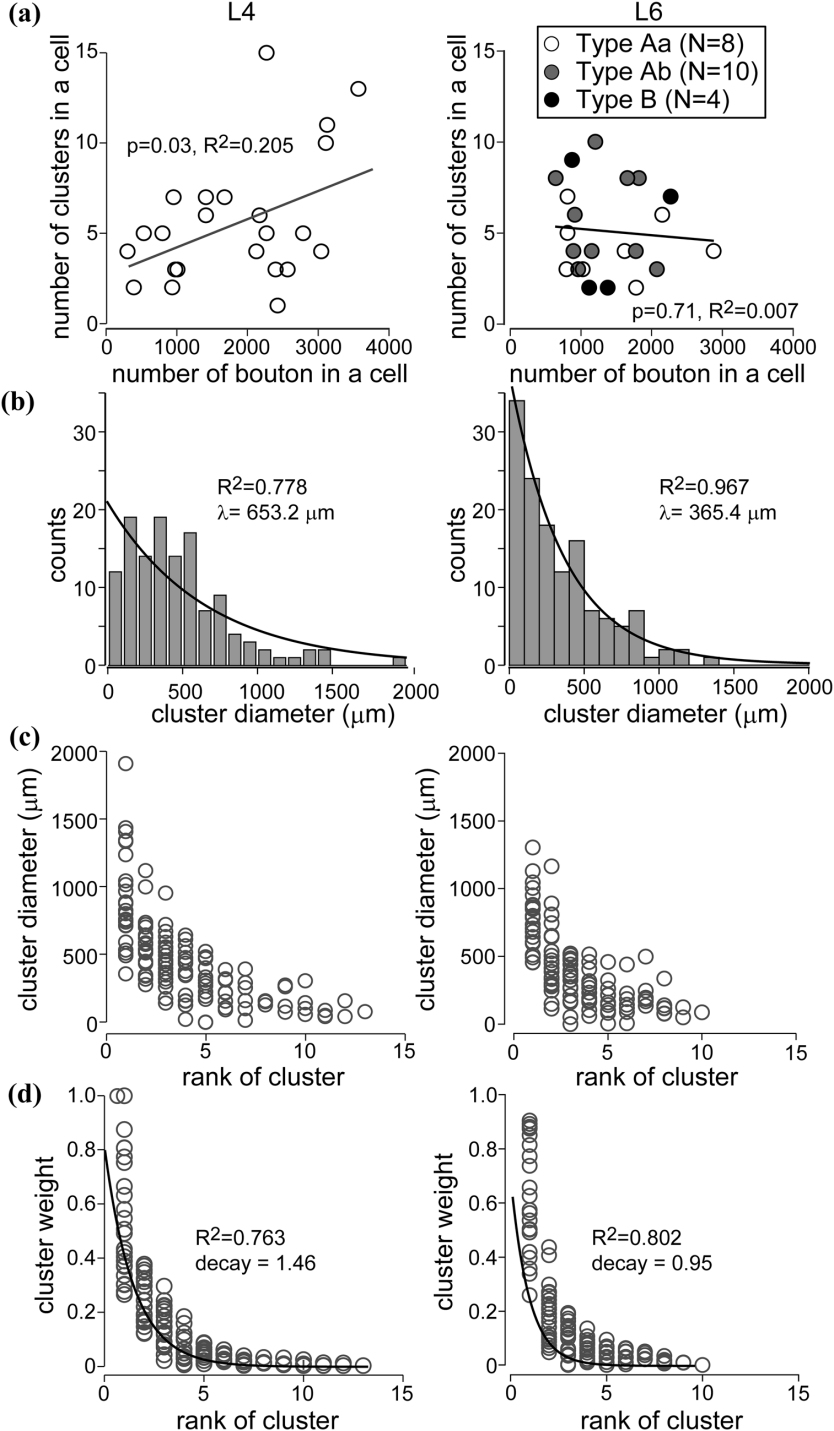
Morphological properties of bouton clustering. For comparison, data on L4 cells were taken from Karube and Kisvárday (2011). **a** Relationship between number of boutons per cell and number of clusters. No significant correlation was observed in L6 cells. In addition, there was no clear difference among cell types. **b** Size distribution of clusters. Compared to L4 bouton clusters, L6 bouton clusters were composed of larger proportion of small clusters (especially <100 μm), resulted in short decay constant in L6. **c** Cluster diameter plotted against rank of clusters. Clusters larger than 500 μm were observed almost in rank 1 and 2 clusters for L6. **d** Cluster weight plotted against rank of clusters. Decay constant was smaller for L6, reflecting cluster weight was steeply decreased in higher rank clusters

Here we examined quantitatively the clustering features of boutons of L6 cells in order to better understand how L6 connections are distributed in the cortical circuitry. To this end, we used the mean-shift algorithm of Binzegger et al. (2007) that had been adopted also for the analysis of layer 4 cells (Karube and Kisvárday 2011). Therefore, the L6 results presented here are viewed in the context of clustering features of L4 spiny cells which not only share the very same thalamocortical input with L6 (Gilbert and Kelly 1975; Gilbert and Wiesel 1979; Lund et al. 1979; Martin and Whitteridge 1984), but also receive a substantial drive from L6 axons (McGuire et al. 1984; Somogyi 1989; Ahmed et al. 1994). The analysis revealed clusters of boutons in all but one L6 cell. Interestingly, the number of clusters per cell did not differ between L4 and L6 cells (5.9 ± 3.5 and 5.1 ± 2.4, respectively; Fig. 4a) neither was there a difference among L6 cell types. Contrary to L4 cells, there was no significant correlation between the total number of boutons and the total number of clusters of L6 cells (Fig. 4a; *p* = 0.71). Bouton clusters of L6 cells were commonly small in size (Fig. 4b, c). The proportion of small bouton clusters (<100 μm diameter) was larger for L6 than for L4 cells (L6: 34 of 112 clusters vs L4: 12 of 130 clusters; *p* < 0.01). Size distribution of the clusters followed an exponential function with a spatial decay constant of 365.4 mm for L6 and 653.2 mm for L4, indicating the occurrence of larger clusters for L4 cells (Fig. 4b). The relative strength or “weight” of the bouton cluster was quantified as the proportion of boutons in individual clusters to all boutons. The bouton cluster possessing the largest number of boutons, namely, “carrying” the heaviest weight, was called the rank 1 cluster. In comparison with L4 cells rank 1 clusters of L6 cells tended to possess higher weight than those of L4 cells, although the difference was not significant (Fig. 4d). However, the weight of the rank 2 clusters steeply decreased and the ratio of weight of the rank 2 cluster to that of the rank 1 cluster was significantly lower in L6 cells than in L4 cells (0.38 ± 0.27 vs 0.59 ± 0.30; *p* < 0.01). The exponential fit of these plots revealed a smaller decay constant for L6 clusters than for L4 ones (Fig. 4d; 0.95 and 1.46, respectively). The spatial distribution of bouton clusters also differed between L4 and L6 cells. Rank 1 clusters were more proximally located in L4 cells than L6 cells (*p* = 0.028; Supplementary Fig. 5a). Rank 1 clusters were more proximally located than rank 2 clusters in L4 (*p* = 0.00008), but not significant in L6 cells (*p* = 0.09). This arises from the difference of vertical bouton distribution, since 2D distance from the soma to rank 1 clusters was not significantly different between L4 and L6 cells (Supplementary Fig. 5b, c). Two-D distance to rank 1 clusters was smaller than to rank 2 clusters in both L4 (*p* = 0.0005) and L6 (*p* = 0.032) cells (Supplementary Fig. 5b). The above results indicate that the L6 rank 1 clusters dominate in power and the remaining clusters are relatively small in size and number of boutons.

### Orientation distribution of boutons

One of the main goals of this study is to determine how boutons of L6 axons are distributed in orientation maps. Because the intrinsic signals to the orientation map derived mainly from superficial layers of the cortex (estimated layer 4 and above; see Tian et al. 2011) and that L6 cells examined here were located at least 1 mm below this zone, a set of strict criteria had to be imposed as to which of the labeled cells are selected for this study. Along this line we sought evidence to clarify that RF properties were preserved through the entire cortical thickness including L6. Unit recordings were examined in the area where optical imaging was carried out and found that only a subtle shift of the RF center position along the same recording track perpendicular to the cortical surface (average: 1.56° ± 2.38°, *N* = 71 penetrations) was present. Orientation preference derived from imaging showed a good match with that of unit recordings (average difference is −2.6° ± 32.4°, *N* = 65 penetrations; Supplementary Fig. 6).

**Fig. 5:**
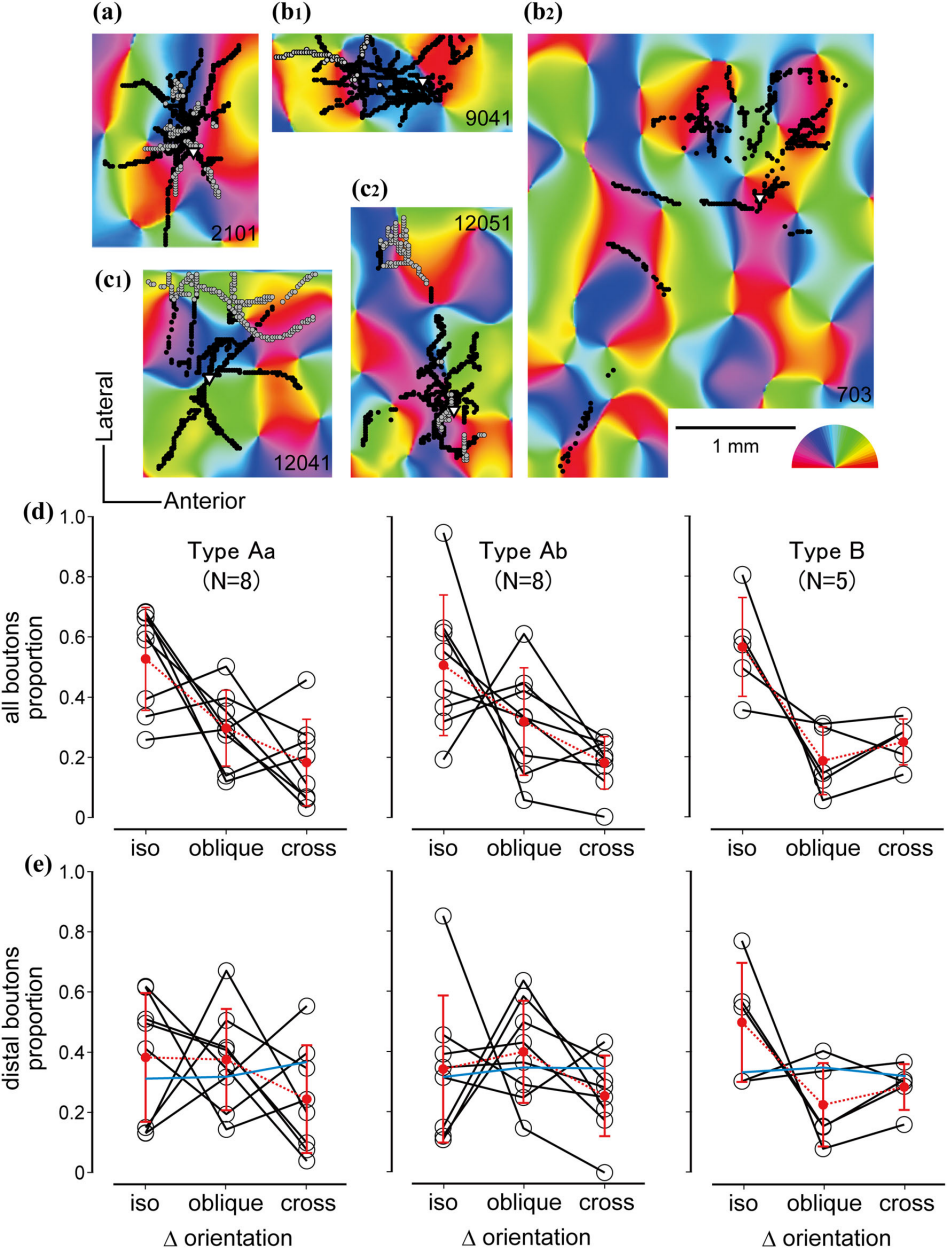
Distribution of boutons of L6 spiny neuron types to orientation map. *Black dots* represent lower tier boutons, *black edged gray dots* upper tier boutons. *Triangle* shows soma position. **a** Type Aa cell located near to a rapidly changing orientation zone. **b**_**1**_ Type B cell located in an orientation domain representing nearly vertical orientations. Most boutons occupy similar orientations to that of the parent soma. **b**_**2**_ Another Type B cell, whose axons extended widely showing chiefly iso-orientation preference. **c**_**1**_ Type Ab cell projecting to a broad range of orientations. **c**_**2**_ Type Aa cell with boutons preferring similar orientations. *Scale bar* 1 mm. Preferred orientation is indicated by *color code*. **d** Orientation preference of boutons for all 21 spiny cells examined. Difference of preferred orientation between soma pixel and bouton pixel was calculated (∆orientation) and binned into iso (<30°), oblique (30°–60°), and cross-(>60°) orientations. *Each black line* represents a single cell. *Red line* represents the mean, *error bars* indicate SD. **e** The same analysis as shown in **d** was applied to distal boutons alone defined as >250 μm away from the parent soma. Note that iso-orientation preference is strongest for Type B. *Blue line* indicates the mean orientation distribution of all pixels within the area of distal axons (see “Materials and methods”). Importantly, pixel distributions showed an almost equal representation in all orientation categories indicating the absence of a “forced choice” situation

The relationship between single cell bouton distribution and functional maps was analyzed for 21 out of 23 reconstructed cells. Two Type Ab cells (#12062 and #1503 in Figs. 1, 2) were omitted from the analysis because >30 % of their boutons were found outside of reliable map regions, i.e., close to edge of the mapped area representing the bony rim of craniotomy. For the sake of simplicity, boutons were assigned in two depth categories: an upper cortical tier comprising L1–L3 and lower cortical tier comprising L4–L6. Distribution of boutons in upper and lower cortical tiers on orientation maps are illustrated in Fig. 5a–c. In addition to this the output selectivity in terms of orientation was determined for each labeled cell. To this, the orientation difference between the parent soma and each bouton was computed pixel-by-pixel (∆ori, range 0°–90°). For the sake of simplicity and comparing the findings with previous data on single cell connections, ∆ori values were divided into iso (<30°), oblique (30°–60°), and cross (>60°) orientation categories and the proportion of iso-, oblique-, and cross-orientation preferred boutons graphed for each cell (Fig. 5d). The mean proportion of iso-, oblique-, and cross-orientation preferred boutons for all 21 cells of L6 was 52.6 ± 18.8, 27.7 ± 14.9 and 19.7 ± 10.9 %, respectively, suggesting an iso-orientation bias similarly to earlier reports for layer 4 (Yousef et al. 1999; Karube and Kisvárday 2011) and other layers (Bosking et al. 1997; Kisvárday et al. 1997; Schmidt et al. 1997; Sincich and Blasdel 2001; Chisum et al. 2003). We also compared the proportion of iso-, oblique-, and cross-orientation preferred boutons of L6 cells with that of L4 (*N* = 23 cells; for details see Karube and Kisvárday 2011) and L3/5 [*N* = 6 cells (Buzás et al. 2006)]. Interestingly, when single cells were compared with each other, no significant difference was found between layers (ANOVA). However, when the single cell data were summed for each layer, L3/5 boutons showed a stronger iso-orientation preference compared to L4 (*p* < 0.01 by Fisher’s exact test with *p* value correction) or L6 (*p* < 0.01), probably due to accumulation of small differences.

**Fig. 6:**
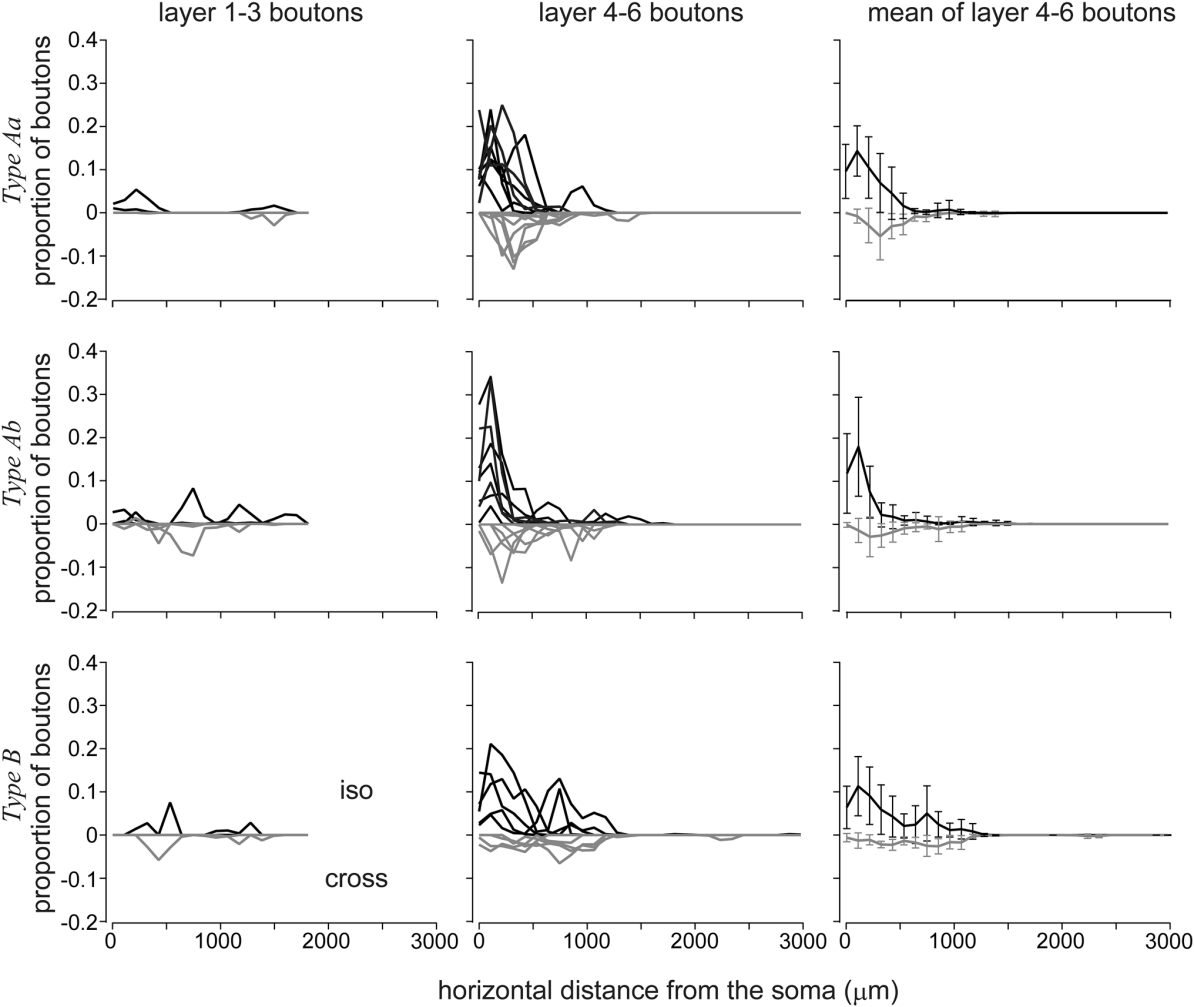
Diversity of orientation preference of boutons as a function of lateral distance from the parent soma. Positive values (*black line*) represent iso-orientation preferred boutons, negative values (*gray line*) represent cross-orientation preferred boutons. *Left column* shows data for upper tier boutons (layers 1–3). For the few cells projecting into upper tier, large cell-to-cell variance in iso- vs cross-orientation distribution can be observed with no clear orientation preference. Middle column shows data for lower tier boutons (layers 4–6), for all three types. For Type Aa and Ab, iso-orientation preferred boutons are most frequent proximal to the soma region (<500 µm) and cross-orientation preferred boutons had a peak only a few hundred μm lateral to the iso-orientation peak. Their deep layer boutons steeply decreased in number over 500 μm. Contrary to Aa and Ab, Type B lower tier boutons produced two peaks of iso-orientation preferred boutons, one near to soma location and one around 700 µm. For Type Ab and B a second smaller peak of cross-orientation preferred boutons was present at about 1000 µm lateral to soma location. Right column represents the mean and SD of deep layer bouton distributions

Comparison of orientation preference of boutons between L6 cells showed that slightly more than half of Type Aa (52.5 ± 29.5 %) and Ab (50.4 ± 23.3 %) boutons occupied iso-orientations largely due to the narrow and columnar arrangement of the axonal field (Figs. 3, 6). The strongest iso-orientation preference was found for Type B (56.5 ± 18.7 %) despite their horizontally wide axonal fields. However, there was no statistical difference between the average iso-orientation preferences of the different types (ANOVA). In order to check whether iso-orientation preference of boutons occurred by chance, for example, due to biased orientation representation of the cortex within the axonal extent of a given cell (“forced choice”), we compared the orientation distribution of boutons with that of orientation map pixels located within a circle that contained the axon of the cell and was centered to the soma location. For each cell, circle radius was set to the horizontal extent of the axon. Comparison between the two distributions showed significant difference (*p* < 0.05 by Fisher’s exact test), suggesting that the orientation distribution of boutons was not by chance. On the other hand, orientation distribution was also measured for only distal boutons, which were defined as boutons further than 250 μm away from the parent soma (outside of the dendrites field). As shown in Fig. 5e, generally iso-orientation preference decreased since local boutons (<250 μm from the parent soma) were located in the home orientation column (Fig. 5e, red lines). For all types, still the mean proportion of iso-orientation preferred boutons was significantly larger than that of iso-orientation pixels located in the axonal field (blue line in Fig. 5e; *p* < 0.01 by Fisher’s exact test), although especially Type Aa and Ab the mean orientation distribution of distal boutons was seemingly close to flat.

Next, we compared the distribution of iso- and cross-orientation preferred boutons with regard to laminar location and horizontal extent from soma origin. To investigate this point, the proportion of iso- and cross-orientation preferred boutons was plotted for the two main cortical tiers, upper (L1–L3) and lower (L4–L6) tiers, as a function of lateral separation from soma location (Fig. 6). For lower tier boutons (*N* = 21 cells), iso-orientation preference was dominant near to the parent soma (<200 μm lateral), although a substantial proportion of boutons showed cross-orientation preference for Type Aa and Ab cells. For the same cell types, at larger lateral distances, (>250 μm, i.e., beyond lateral extent of their basal dendrites), the proportion of iso-orientation preferred boutons decreased steeply until the proportion of iso- and cross-orientation preferred boutons were near equal, implying an orientation-independent distal projection. On the contrary, for Type B cells, the proportion of iso-orientation preferred boutons remained higher than cross-orientation preferred boutons across long horizontal distances (>1 mm from soma; right column). In all cells, upper tier boutons were less numerous than lower tier boutons. For the 15 cells, which had axon collaterals in upper tier, an average of 391 ± 323 boutons per cell was found, while remaining 8 cells did not have upper tier boutons. The proportion of upper tier boutons to all boutons of the respective cell type was 4.8 % for Type Aa, 21.2 % for Type Ab, and 28.7 % for Type B (average 17.1 % of all boutons, range 0–57 %). It should be also mentioned that only those cells were included in this analysis which had >70 % of their upper tier boutons in the reliable map region (2 of Type Aa, 3 of Type Ab, and 2 of Type B). For these upper tier boutons no clear orientation bias could be found probably due the small sample size of the available boutons. Another point that needs to be mentioned here is that oblique-orientations (omitted for clarity of graphs in Fig. 6) had consistently represented mean values between those of iso- and cross-orientation.

### Convergence of boutons from nearby cells

**Fig. 7:**
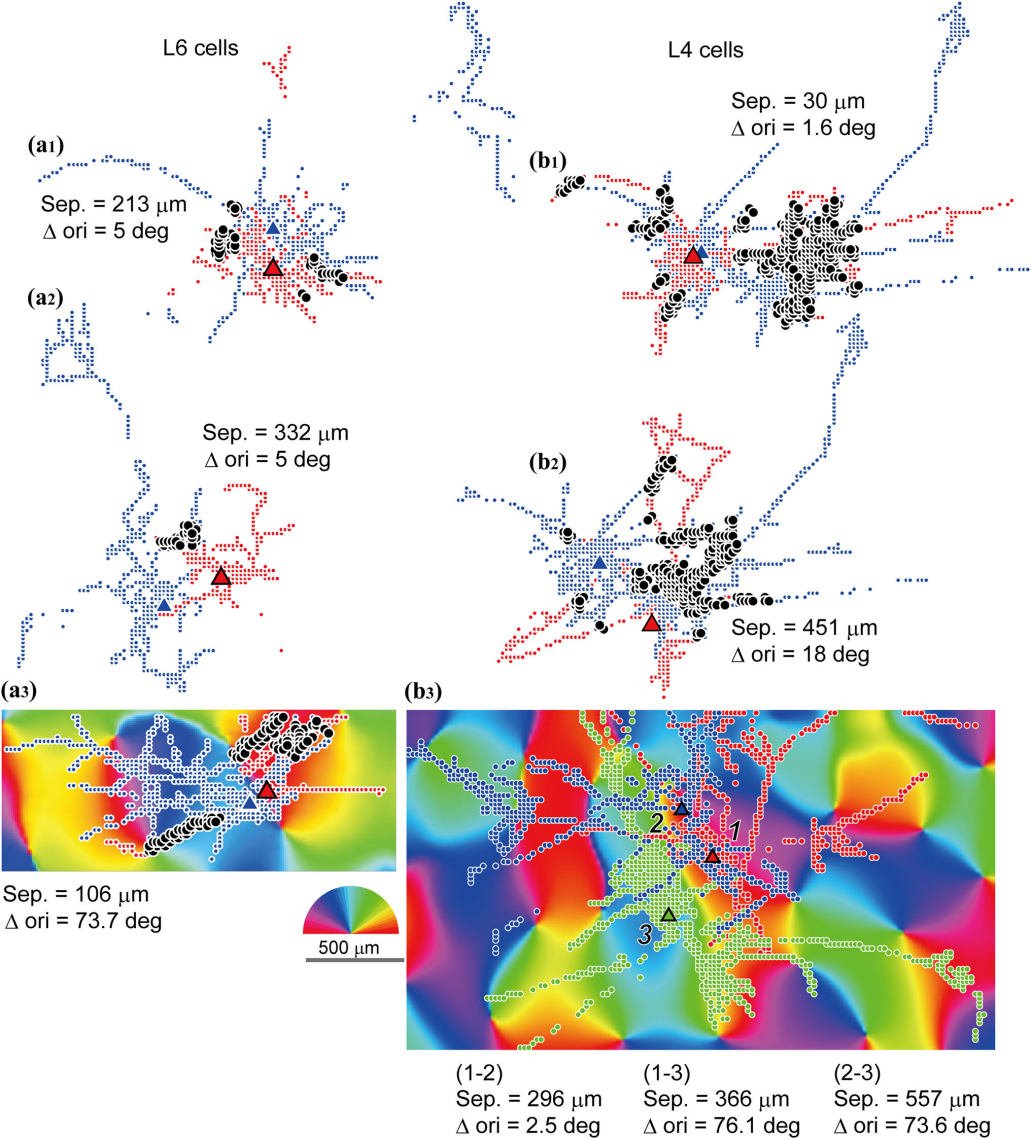
Examples for spatial overlap between boutons of pairs of L6 and L4 spiny cells. **a**_**1**_, **a**_**2**_ Boutons (*red* and *blue dots*) and somata (*triangles of the same color* as the boutons) of nearby L6 neuron pairs possessing similar orientation preference. The bouton distributions of neighboring cells show spatial overlap at just the same image pixel (*black dots*). As shown in Figs. 8 and 9, overlapping pixel positions are shown only for distal boutons defined as >250 μm away from the parent soma. **a**_**3**_ Boutons (*red* and *blue dots*) and somata (*triangles*) of two neighboring L6 cells are superposed on orientation map. Although the cell bodies are located at different orientation preference domains bouton overlap is still observable (*black dots*). **b**_**1**_, **b**_**2**_ Pairwise comparison of neighboring L4 cells possessing similar orientation preference with each other. The axons are elongated chiefly toward similar directions and the distal boutons overlap to a large extent (*black dots*). **b**_**3**_ Three neighboring L4 cells. Somata of cells 1 and 2 occupy similar orientations (*red* and *blue triangles*), whereas soma of cell 3 has a different orientation preference (*green triangle*). Cell 1 and 2 shows a relatively high bouton overlap with each other, whereas cell 3 has only a small overlap with the others, although soma separation between cell 3 and 1 is similar to that of cell 1 and 2 (overlap is not shown). *Sep.* soma separation, *∆ori* difference of preferred orientation. *Scale bar* 500 µm

**Fig. 8:**
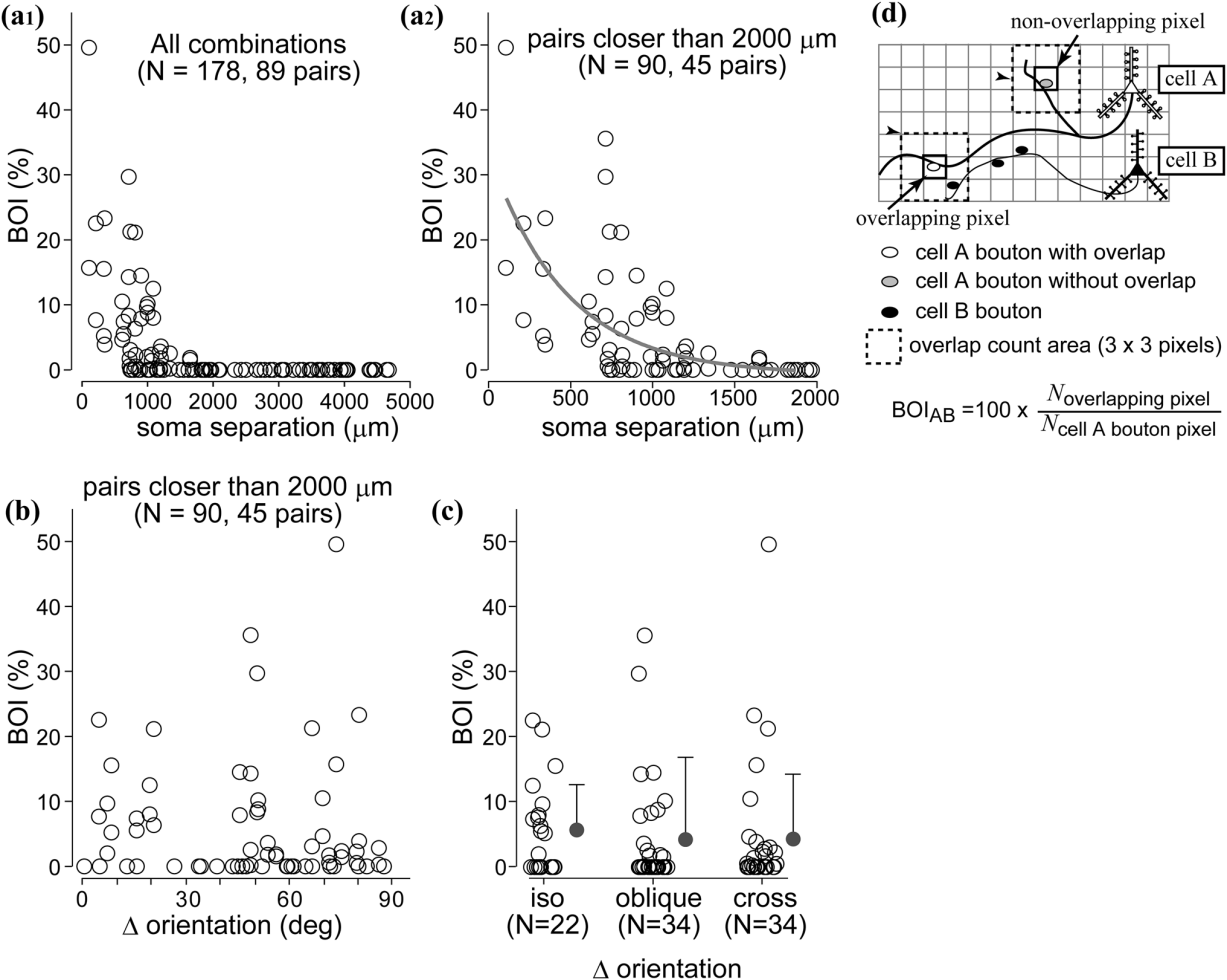
Bouton overlap of L6 cells is dependent on soma separation but independent of ∆ori. Relationship between bouton overlap and soma separation for L6 cell pairs. **a**_**1**_ BOI was higher for closer pairs whereas for larger soma separation (>2 mm), no bouton overlap was observed. The definition of BOI is shown in d (see also “Materials and methods”). **a**_**2**_ The same data as in **a**_**1**_, except that pairs with larger than 2 mm separation are excluded. BOI was negatively correlated with soma separation. *Gray curve* represents fitted exponential (*R* ^2^ = 0.29, *p* < 0.00001, *N* = 90, decay constant of the fit: 472 µm). **b** BOI was independent of orientation difference (∆ori) of parent somata (*R* ^2^ = 0.002, *p* = 0.68). **c** BOI did not differ among iso-, oblique-, and cross-orientation pairs (*p* = 0.11 for iso vs oblique; *p* = 0.19 for iso vs cross; *p* = 0.22 for oblique vs cross). **d** Calculation of BOI. For a pair of cells, A and B, BOI_A→B_ and BOI_B→A_ were calculated separately, which typically resulted in two different BOI values. In this illustrated case, BOI_A→B_ = 50 % and BOI_B→A_ = 33 %. Each pixel corresponds to 21.28 × 21.28 μm^2^

**Fig. 9:**
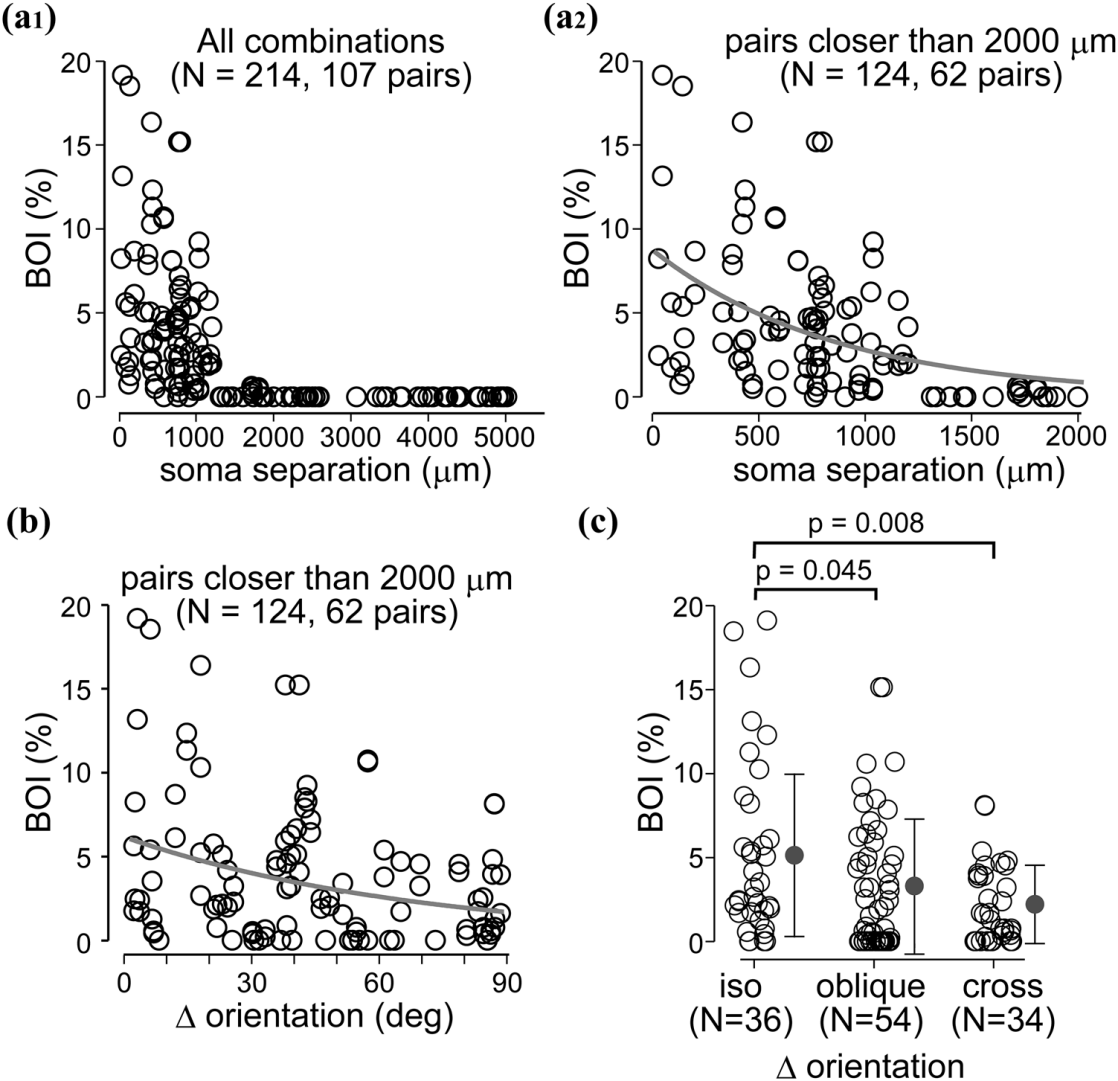
Bouton overlap of L4 cells depends on soma separation and ∆ori. Similar to L6 cell pairs, soma separation and bouton overlap index (BOI) for L4 cell pairs show negative correlation. **a**_**1**_ For larger than 2 mm soma separation, almost no overlap can be seen. **a**_**2**_ Same data as in A_1_, except that pairs with larger than 2 mm separation are excluded. *Gray curve* represents fitted exponential (*R* ^2^ = 0.26, *p* < 0.0001, *N* = 124, decay constant of the fit: 865 µm). **b** BOI also showed a reverse correlation with ∆ori of parent somata (*R* ^2^ = 0.08, *p* = 0.002, *N* = 124, decay constant of the fitted exponential is 70.1°). **c** Cell pairs preferring similar orientations (∆ori ≤30°, iso) showed significantly larger BOI than those with orientation difference falling in oblique (30° < ∆ori ≤ 60°) or cross-orientation (60°< ∆ori) categories

In some cases for L6 cells (UD009, UD012 and UD021; Supplementary Fig. 2) and some cases for L4 cells (UD007, 008 and 009; see Supplementary Fig. 2 in Karube and Kisvárday 2011), reconstructed spiny cells were located so close to each other that some of their boutons were found in the same image pixels. Such a spatial overlap of boutons emitted by neighboring cells implies potential convergence of axons terminating in the same orientation column or, as cannot be excluded, on a common target, such as a single dendrite. It should be added here that our tissue sample contained closely labeled cells not only in layer 6, but also in layer 4. Hence our analysis was extended for both thalamic input-recipient layers. Figure 7a shows such closely located L6 cells. The somata of nearby cells were located in image pixels which had similar orientation preferences (Fig. 7a_1_, a_2_). Their boutons not only occupied image pixels of similar orientations to each other’s but often terminated in the very same pixel position (black dots in Fig. 7a). When, however, neighboring somata were located in pixels with different orientation preference, their boutons showed overlap in some cases (Fig. 7a_3_). A similar tendency was also observed for L4 cells (Fig. 7b; see also Karube and Kisvárday 2011 for detailed morphology of L4 cells.). In order to uncover neighborhood relationship of boutons, first the orientation preference of converging boutons was determined. Next, we introduced a bouton overlap index (BOI, see “Materials and methods” and Fig. 8d) which represents the proportion of overlapping distal boutons between a pair of cells and informs about convergence and potential interaction at those locations. BOI returns a value of zero for no overlap and 1 for a complete overlap. Regarding L6 cell pairs (89 combinations of a pair of cells; chiefly Type Aa and Ab), BOI had the highest values primarily for those cell pairs whose soma separation was less than 1.5 mm and, within this range, showing an obvious negative correlation (Fig. 8a). Then, the orientation distribution of pixels containing overlapping boutons was determined and compared with orientation distribution of pixels representing the parent somata. In this regard, if cells with similar orientation preference tend to innervate similar cortical location, BOI and orientation difference between somata (∆ori) should negatively correlate. However, the data showed no significant correlation (Fig. 8b) indicating that the BOI values of iso-, oblique-, and cross-orientation cell pairs do not differ significantly from each other (Fig. 8c). The findings are compatible with a quasi-orientation independence of the L6 intrinsic circuitry showing only a weak orientation selectivity of distal connections, especially, of those of Type Aa and Ab cells. This aspect of orientation preference is also interesting to investigate for the cell population of L4 which has an intimate relationship with L6, such as being one of its main synaptic targets and receiving input from the same thalamocortical input fibers as L6. For 23 L4 spiny cells containing spiny stellate and star pyramidal cells (for details, see Karube and Kisvárday 2011), 107 combinations of cell pairs were analyzed. Figure 9a shows the relationship between BOI and soma separation for L4 cell pairs, which was similar with that of L6 cell pairs (cf. Figure 8a). In contrast to the L6 case, ∆ori of L4 cells negatively correlated with BOI (Fig. 9b). For a simple demonstration of the latter relationship, ∆ori was binned into iso-, oblique-, and cross-orientations. The resulting graph clearly showed that iso-orientation preferring cell pairs are associated with significantly higher BOI values than non-iso-orientation preferring cell pairs (Fig. 9c). The index BOI indicated frequency of pixels possessing convergent boutons and did not reflect number of boutons. To represent strength of convergence, the number of boutons in convergent pixels was counted (Supplementary Fig. 7). For L6 cells, strong convergence, i.e., occurrence of large number of boutons in a pixel, was more frequent among iso-orientation pixels, and also among those with shorter separation (Supplementary Fig. 7a). In addition, with regard to distance from the parent soma to converging pixels, strong convergence occurred more frequently within 500 μm form the parent soma (Supplementary Fig. 7b). On the contrary, almost little or no convergence was observed between 500 and 1000 μm from the parent soma, but around 1000 μm away from the soma, strong convergence appeared again. For L4 cells, the relationship between strength of convergence and soma separation or ∆ori was not obvious because strong convergence occurred at almost all ranges of ∆ori (Supplementary Fig. 7c, d). A likely explanation is that the cell population of L4 comprises both iso-orientation preferred cells and cross-orientation preferred cells (Karube and Kisvárday 2011).

## Discussion

**Fig. 10:**
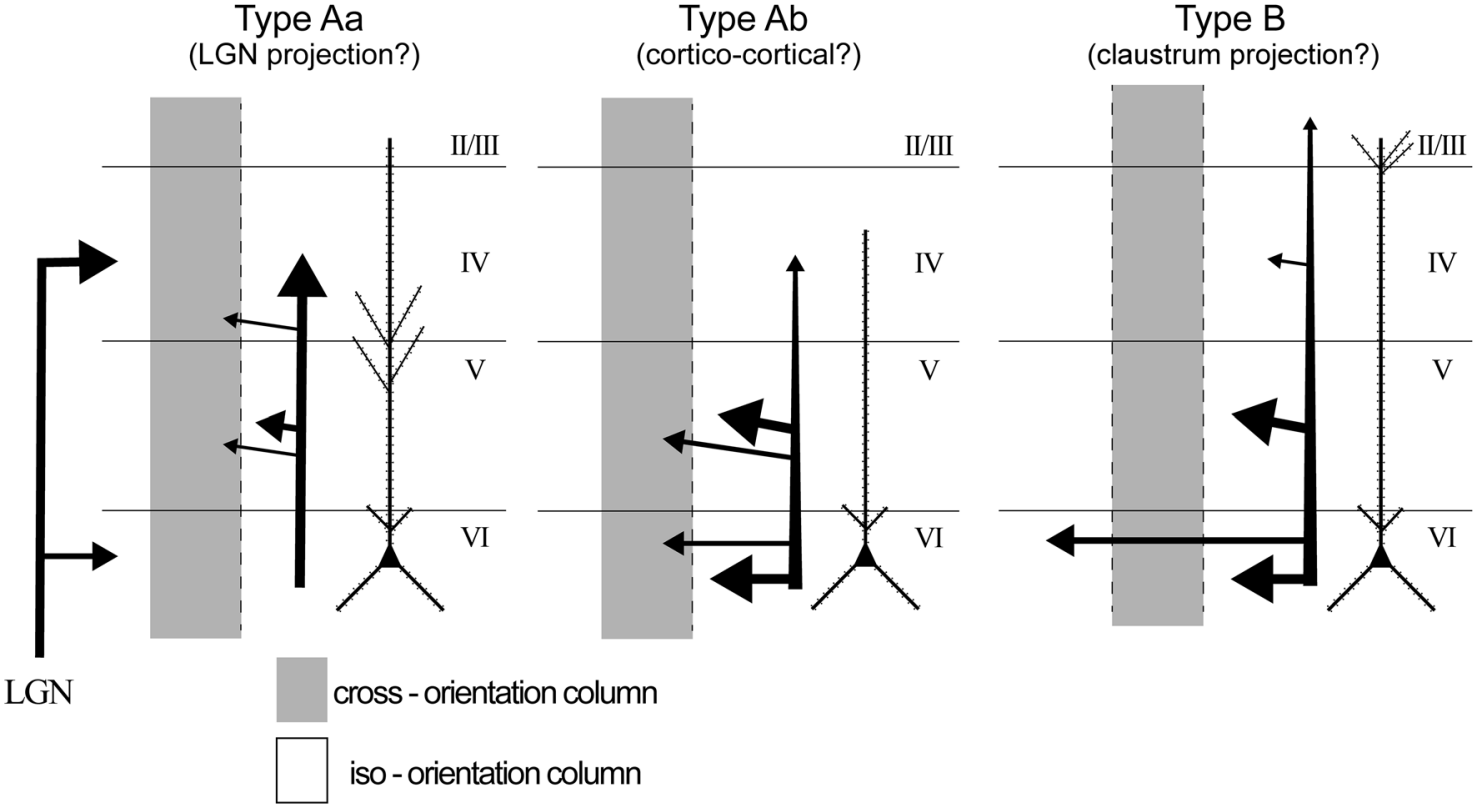
A summary chart showing the main projection features of the three L6 cell types. *Gray shading* represents columns possessing different orientation preference from that of the home column of L6 cells. *Thickness of arrows* and *arrowheads* represent strength of axonal connection. *LGN* lateral geniculate nucleus. The projection target of each Type was not identified experimentally only assumed by taking into account data of previous reports (see “Discussion“)

We analyzed the orientation distribution of boutons for three morphological types of L6 spiny cells and showed that (1) L6 cells could be classified based on dendrite morphology (Figs. 1, 2), i.e., cell Types Aa, Ab and B; (2) horizontal and vertical distribution of intra-cortical axon correlated with dendritic cell types (see Fig. 3). Type Aa axon was more columnar and dense in upper cortical tiers, whereas Type B axon distributed horizontally wider; (3) orientation preference of boutons often differed between upper and lower tiers of which lower tier boutons showed a stronger bias towards iso-orientation preference than upper tier boutons (Fig. 6); (4) distal projection of L6 cell types targeted typically a broad range of orientations with the exception of Type B (putative claustrum projecting cells, see below) which showed relatively strong iso-orientation preference (Figs. 5, 6); and (5) bouton projection overlap of nearby L6 cells depended on soma separation but not on orientation preference (Fig. 8) contrary to L4 spiny cells whose bouton overlap depended both on soma separation and orientation preference (Fig. 9). In the following we will discuss L6 cell types in the light of earlier reports and consider their putative functional contribution in visual processing (see also Fig. 10). We will also compare their projection characteristics with those of L4 cells as being both cell populations the main recipient of visual thalamic inputs.

### Methodological considerations

L6 is the deepest and often the thickest layer where columnar organization—both functional and structural—can be disturbed by two main factors: closeness of the underlying white matter and curvature of the cortex. In a flat cortical region fiber bundles of the white matter enter the grey matter typically perpendicular to the cortical surface. However, curvature of the cortex can distort columnar organization in deep layers rendering any functional and structural interpretation difficult here. This is particularly true for interpreting intrinsic signals, which derive largely from upper cortical layers therefore extrapolating such signals to L6 has to be treated with caution. Primary visual cortex of the cat is situated chiefly along the lateral gyrus, which comprises a centrally located rostro-caudally elongated flat zone on the top of the gyrus. Here, the columnar organization of the cortex is rather regular as confirmed by unit recordings across all layers (see “Materials and methods”; Murphy and Sillito 1986; Karube and Kisvárday 2011). We used only this flat zone for labeling L6 spiny cells to investigate correlation of bouton distribution with orientation map.

The reconstructed cells studied here were labeled using extracellular (see “Materials and methods”) tracer injections that raises the question as to completeness of the labeling. We used only those cells which showed strong labeling in the soma and the dendrites as well as the axons to their ends. However, a common drawback of extracellular labeling is that some of the labeled axon collaterals cannot be traced due to the high density of other labeled structures at the center of the injection site. We assume that this feature can explain why the number of boutons in our L6 sample (mean 1399 ± 584; range 640–2878) is smaller than expected on the basis of intracellularly labeled L6 cells for which all axons can be readily traced (Binzegger et al. 2004). Another explanation could be that our cell sample simply lacked certain types of L6 cell possessing large number of boutons. However, the latter explanation is unlikely because the mean dendritic length of both apical and basal dendrites (8.34 ± 2.49 mm, range 5.54–17.86 mm) of our L6 sample was almost the same as that reported by Binzegger et al. (2004) indicating that both studies analyzed comparable cell populations.

### Types of L6 spiny cells

The morphological features of the L6 cell types analyzed here are consistent with previously published data obtained in the cat (Gilbert and Wiesel 1979; Lund et al. 1979; Martin and Whitteridge 1984; Katz 1987; Lorente de No 1992; for review see Briggs 2010; Thomson 2010). With regard to the target regions L6 excitatory cells are heterogeneous of which about 50 % project into the LGN, >10 % into the claustrum and about 20 % are cortico-cortical (Gilbert and Kelly 1975; Harvey 1980a, b; LeVay and Sherk 1981; McCourt et al. 1986; Katz 1987; Hirsch et al. 1998). The above L6 types differ not only in their projection target areas, but also can be distinguished on the basis of morphological features such as dendritic branching pattern and axon distribution. Katz (1987) used the Sholl analysis for comparing dendritic morphology of pyramidal cells projecting into different target areas. Although no statistical values were reported, the results revealed minor differences between projection types (see Fig. 17 in Katz 1987). Here we also employed the Sholl analysis and found subtle differences for basal dendrites but only at the proximal parts (*p* < 0.05 between Type Aa and Type B, and between Type Aa and Type Ab at the range of 10–30 μm form the soma, paired Wilcoxon test with compensation for multiple comparisons). Apical dendrites differed significantly (*p* < 0.05) among types at the ranges of 70–190, 310–410 and 720–780 μm from the soma (Supplementary Fig. 3). A possible interpretation of the morphological similarity of basal dendrites among types is that they sample a rather similar input, whereas apical dendrites are exposed to a wide range of input of which each type is engaged with a particular set of inputs.

In addition to morphological attributes L6 cells show rather distinct physiological characteristics: most LGN projecting L6 cells have simple RF characteristics (Gilbert 1977; Henry et al. 1979; Harvey 1980a, b; Martin and Whitteridge 1984; Grieve and Sillito 1995; Hirsch et al. 1998), although a subpopulation of them display complex RF features and additionally project into the extrageniculate and perigeniculate nuclei (Tsumoto and Suda 1980; Murphy and Sillito 1996; Murphy et al. 2000; Jones 2007). Claustrum projecting cells are typically monocular and have simple-like RFs with extreme length (10° or longer, Grieve and Sillito 1995). On the basis of distinct morphological signatures, here we make an assumption that our Type Aa corresponds to LGN projecting simple cells and Type B to claustrum projecting cells. On the other hand, Type Ab shares features with those cortico-cortical projecting cells which typically show complex RFs structure and restrict their lateral connections chiefly to infragranular layers (Hirsch et al. 1998). Hence, it is deemed that our cell sample represents the major neuron types of the otherwise highly heterogeneous L6 cell population (for reviews, Briggs 2010; Thomson 2010).

For depth distribution of our L6 cell samples, there was no significant difference among the three types. In the primate and the tree-shrew visual cortex, L6 can be subdivided into zones which parallel the termination of subcortical input fibers (Hubel and Wiesel 1972; Lund and Boothe 1975; LeVay and Gilbert 1976; Blasdel and Lund 1983; Fitzpatrick et al. 1983; Braak 1984; Conley et al. 1984; Usrey and Fitzpatrick 1996). In the cat, such a stratification of the L6 circuitry is still elusive. The distribution of VGluT2-positive terminals within L6 was only slightly different across the full depth of L6 (Supplementary Fig. 8) and does not support a clear stratification within L6. Altogether, the distribution of our reconstructed cells corroborate results of previous studies in finding no marked difference between the cell population of upper and lower subzones of L6 (Lund et al. 1979; McCourt et al. 1986). Contrary to anatomical data the few functional studies that are available pointed out the existence of subzones within L6. Hirsch et al. (1998) reported that deep zone of L6 contains mainly complex cells, while the study of Bullier and Henry (1979) showed that the proportion of complex cells is moderately larger in the upper half of L6 than in the lower half. Obviously, to disclose whether neurons of the same or similar phenotype may possess distinct functional properties and whether they are organized in distinct subzones of L6 further studies are necessary.

### Comparison of bouton clustering between L6 and L4 cells

Clustering of boutons is considered as a fundamental property of most cortical neuron types (Binzegger et al. 2007; Muir et al. 2011). Here we made a quantitative comparison between bouton cluster parameters on a relatively large sample of L6 and L4 spiny neurons (see Table 2). As described above, many of the bouton cluster parameters such as spatial extent, cluster rank and weight and their relationships were quite similar for L4 and L6 spiny cells and only in a few parameters they differed significantly (Fig. 4, Supplementary Fig. 5; Table 2). Among these a notable difference was that rank 1 clusters of L4 had a smaller lateral displacement from the parent soma and rank 2 clusters had a larger displacement from the parent soma than the clusters of L6. Nonetheless, these subtle differences in the spatial organization of bouton clusters may explain the less patchy character of L6 axons compared to L4 axons and that a single bouton cluster of the L4 cell can occupy neatly either iso- or cross-orientation domains (Karube and Kisvárday 2011), a feature that is not seen for L6 cells. As for the latter difference, at present, there is no obvious explanation. It can, however, be speculated that while L4 cells commonly contribute to setting up orientation components of RFs instead L6 are engaged in less orientation-dependent duties (see below).

**Table 2:** Basic properties of bouton clusters in layer 4 and 6 pyramidal cells

	Layer 4	Layer 6	Difference
Cell number	22	22	
Number of total clusters	130	112	
Cluster number/cell	5.9 ± 3.5	5.1 ± 2.4	
Cluster diameter	442.5 ± 328.2	394.2 ± 283.7	
Cluster weight	0.169 ± 0.199	0.196 ± 0.246	
Distance from the soma to the cluster center	850.0 ± 574.6	711.2 ± 439.0	L4 > L6*
1st rank cluster			
Weight	0.512 ± 0.195	0.629 ± 0.208	
Distance	316.4 ± 337.3	520.9 ± 423.4	
Diameter	900.8 ± 366.8	771.7 ± 226.3	
2nd rank cluster			
Weight	0.253 ± 0.085	0.191 ± 0.105	L4 > L6*
Distance	777.9 ± 378.1	672.9 ± 319.1	
Diameter	577.0 ± 203.5	491.6 ± 265.8	
Weight 2nd/1st	0.59 ± 0.30	0.38 ± 0.27	L4 > L6*

Values represent mean ± SD. Statistical significance was examined by Mann–Whitney *U* test (* *p* < 0.05)

### Bouton overlap between neighboring cells in L4 and L6

Bouton overlap is an emerging result of our experimental approach that can be used for estimating to what extent cortico-cortical inputs converge at similar or dissimilar orientations. Here we took advantage of a previous study that had focused on the orientation distribution of L4 spiny cells, some of which also had spatially overlapping axons (Karube and Kisvárday 2011). By employing identical analysis, it is seen that bouton overlap between L4 spiny cells depends weakly on orientation preference (see Fig. 9b, c), whereas bouton overlap between L6 cells does not (see Fig. 8b, c). Since on average, there is a dominance of iso-orientation preferred boutons of L6 neurons almost at all lateral distances from soma location (see right column in Fig. 6), it is surprising that their bouton overlap between L6 cells lacks orientation dependence. However, when the data are inspected in more detail it turns out that bouton overlap is more extensive between cells which are located closer to each other or between those whose parent soma and boutons occupy iso-orientation (Supplementary Fig. 7a). Interestingly this is not the case for L4 cells, which in turn show a bias to overlapping boutons which are far from their parent soma and represent cross-orientation (Supplementary Fig. 7b). The complexity of bouton overlap observed for L6 cells is probably due to different spatial organization of connections established by the different cell types. It is conceivable that Type Aa and Ab axons whose cross-orientation preference increases with distance account for most of the non-iso-orientation biased bouton overlap. On the other hand, this is compensated by an iso-orientation biased bouton overlap typically by Type B cells. Consequently, the L6 population data for bouton overlap show no correlation with orientation preference. Inevitably, it would have been useful to carry out a cell type related quantitative analysis of bouton overlap. However, there was no sufficient number of labeled Type B cells in our sample, hence a cell type-dependent analysis of bouton overlap could not be carried out. Finally, it should be mentioned, that due to geometrical constraints and the distance of L6 from the imaged cortical surface, we cannot exclude the limited accuracy of alignment between the orientation map and the anatomical reconstruction that is inherent to the method used here.

### Functional implications of L6 cell types

As a population, L6 cells showed an iso-orientation bias, similar to other layers (Fig. 5). From the circuitry point of view, L6 cells are implicated at least in two functional streams: the major intracortical input sources to L4 on the one hand and as one of the output sources to subcortical regions, mainly LGN and claustrum, on the other hand. Electron microscopy of L6 cells estimated that they account for 20–40 % of all synapses on L4 spiny stellate cells (Ahmed et al. 1994, 1997; Binzegger et al. 2004; Tanaka et al. 2011; Pichon et al. 2012). In addition to this, L6 outputs were shown to activate inhibitory neurons too (West et al. 2006; Olsen et al. 2012; Bortone et al. 2014), although the relative proportion of the targeted excitatory and inhibitory cells is still a matter of debate (McGuire et al. 1984; Somogyi 1989; Ahmed et al. 1994, 1997; Staiger et al. 1996). Importantly, the functional consequence of these findings is that facilitatory and suppressive inputs must go hand in hand and mutually modify the activity level of the entire L4 circuitry through which they may contribute to emerging novel receptive field properties. Here we showed that Type Aa (putative LGN-projection) cells provide columnarly arranged, chiefly iso-orientation input to L4, and to a lesser extent, Type Ab (putative cortico-cortical) cells also do so (Figs. 3, 5). On the other hand, the same L6 cells can contact a relatively large proportion of cross-orientation sites in L4 whereby sharpening of orientation preference possibly via lateral inhibition can be envisaged (Crook et al., 1991; Crook and Eysel 1992). Another functional interpretation of the relatively rich input from L6 to L4 is not necessarily to affect spatial tuning characteristics such as orientation preference. For example, recent data obtained in rodent visual cortex indicate that L6 cells can improve signal to noise ratio and control synaptic gain in L4 (Olsen et al. 2012; Bortone et al. 2014). It is also conceivable that L6 connections are engaged with temporal characteristics of RFs without changing spatial tuning characteristics as was found in the monkey visual cortex (Ringach et al. 1997). Clearly, the many different modes of operation of how L6 may act on L4 by modifying or even generating novel RF properties (see below) indicate innate sophisticated mechanisms in elaborating orientation-specific excitatory responses of first order L4 cells. Indeed signatures of the above plastic changes, either facilitatory or suppressive were detected in slice preparation showing small amplitude and high variance in the synaptic transmission involved in L6 and L4 cells which, nonetheless, differed between the two projection types, i.e., cortico-cortical vs thalamocortical projecting L6 cells (Stratford et al. 1996; Tarczy-Hornoch et al. 1999; Mercer et al. 2005; West et al. 2006).

Regarding the functional underpinnings of different types of L6 cell the most specific interaction studied so far concerns the generation of length tuning. Inspired by the very long RFs (8°) of a substantial proportion of L6 cells (Gilbert 1977) it was suggested that they are implicated in the generation of length tuning property, i.e., end-inhibition, of “hypercomplex” cells (Bolz and Gilbert, 1986) via L4 inhibitory neurons (see McGuire et al. 1984). However, data from other laboratories casted doubt on the significance of L6 neurons in generating end-inhibition because silencing of L6 by local iontophoretic application of either GABA or muscimol (a potent GABA_a_ receptor agonist) revealed a decreased responsiveness to the optimal, short stimulus in L4 cells rather than an increased responsiveness to non-optimal, long stimulus (Grieve and Sillito 1991a, b). Further support to the latter observations was provided by postembedding GABA-electron microscopy which showed a smaller proportion of L6 boutons terminating on L4 GABAergic dendrites (14 % in cat: in Somogyi 1989; 32 % in rat: in Staiger et al. 1996) than had been reported solely on the basis of ultrastructural features (93 % in cat: in McGuire et al. 1984). The present anatomical findings and those of former intracellular studies (Gilbert and Wiesel, 1979; Martin and Whitteridge 1984; Katz 1987; Hirsch et al. 1998) are, in fact, more consistent with the conclusion reached by Grieve and Sillito (1991a, b). Firstly, Type Aa cells have a spatially restricted projection into L4 and it is tempting to speculate that they represent the population with an RF size too small for exerting length tuning (Grieve and Sillito 1995). Secondly, Type Ab and B cells which do have relatively long horizontal axons and presumably possess long RFs do not typically send a rich input to L4 (see Figs. 2, 3). It is noteworthy that despite the aforementioned contradictions the layer 6–4 circuitry does contain elements which are in line with the notion of a putative role in length tuning. Consequently, for a thorough understanding of the above process future studies shall employ, for example, in vivo pair-recordings of L6 and L4.

As mentioned above an estimated 50 % of L6 cells have unusually long RFs summing to 8 degrees or more (Gilbert 1977; Bolz and Gilbert 1986, 1989; Grieve and Sillito 1995). In this regard a reasonable assumption is that Type Ab and B population providing the longest axons may have the longest RFs. From this it follows that according to the orientation distribution of Type Ab and B axons quite a mixed effect can be elicited on the recipient cells in L5, i.e., local- and distal (>1 mm) axon collaterals could activate iso-orientation sites whereas in the 400–800 μm range also non-iso-orientation sites (see Fig. 5b_2_, 6). Because in the cat visual cortex the targeted L5 cells can project to other cortical layers as well as several subcortical structures (Hübener et al. 1990; Kasper et al. 1994), including superior colliculus (Garey 1965), thalamic nuclei (LeVay and Sherk 1981) and visual striatum (Khibnik et al. 2014), the most obvious scenario is that the above orientationally mixed effect can (through the L5 network) modify the activity of several brain regions.

Of the L6 cell population an estimated 4–10 % is known to project into the visual claustrum where RFs can be as long as 40° (Creutzfeldt et al. 1980; Sherk and LeVay 1981). The present data provide support for a two-step mechanism how these large and orientation-specific receptive fields are established in the claustrum. First, a spatial convergence of long-range axons deriving from L5 and L6 spiny cells onto Type B would generate an RF length that often exceeds 10° or longer. Second, Type B axons, which establish iso-orientation connections more frequently than any other L6 cell types (Fig. 5) forward an iso-orientation based input on claustral cells whereby a several fold increase in RF elongation is achieved in the targeted cells. It remains to be seen by future studies for what type of visual processing the very long RFs are utilized by the claustrum (for review, see Crick and Koch 2005; Smythies 2015).

Taken together the present data revealed a diverse relationship between the different morphological types of L6 cells and orientation selectivity. The integrative nature of L6, being one of the main output layers of the visual cortex and its widespread relation in receiving input from other layers render L6 a versatile stage in processing visual information.

## Electronic supplementary material

Below is the link to the electronic supplementary material.

Supplementary material 1 (DOCX 28 kb)

Supplementary material 2 (TIFF 7533 kb)

Supplementary material 3 (TIFF 2173 kb)

Supplementary material 4 (TIFF 2541 kb)

Supplementary material 5 (TIFF 1756 kb)

Supplementary material 6 (TIFF 1814 kb)

Supplementary material 7 (TIFF 1104 kb)

Supplementary material 8 (TIFF 2652 kb)

Supplementary material 9 (TIFF 8914 kb)

## References

[CR1] Ahmed B, Anderson JC, Douglas RJ, Martin KA, Nelson JC (1994). Polyneuronal innervation of spiny stellate neurons in cat visual cortex. J Comp Neurol.

[CR2] Ahmed B, Anderson JC, Martin KA, Nelson JC (1997). Map of the synapses onto layer 4 basket cells of the primary visual cortex of the cat. J Comp Neurol.

[CR3] Andermann ML, Gilfoy NB, Goldey GJ, Sachdev RN, Wolfel M, McCormick DA, Reid RC, Levene MJ (2013). Chronic cellular imaging of entire cortical columns in awake mice using microprisms. Neuron.

[CR4] Bannister NJ, Nelson JC, Jack JJB (2002). Excitatory inputs to spiny cells in layers 4 and 6 of cat striate cortex. Philos Trans R Soc Lond B.

[CR5] Binzegger T, Douglas RJ, Martin KA (2004). A quantitative map of the circuit of cat primary visual cortex. J Neurosci.

[CR6] Binzegger T, Douglas RJ, Martin KA (2007). Stereotypical bouton clustering of individual neurons in cat primary visual cortex. J Neurosci.

[CR7] Blasdel GG, Lund JS (1983). Termination of afferent axons in macaque striate cortex. J Neurosci.

[CR8] Blasdel GG, Salama G (1986). Voltage-sensitive dyes reveal a modular organization in monkey striate cortex. Nature.

[CR9] Bolz J, Gilbert CD (1986). Generation of end-inhibition in the visual cortex via interlaminar connections. Nature.

[CR10] Bolz J, Gilbert CD (1989). The role of horizontal connections in generating long receptive fields in the cat visual cortex. Eur J Neurosci.

[CR11] Bonhoeffer T, Grinvald A (1991). Iso-orientation domains in cat visual cortex are arranged in pinwheel-like patterns. Nature.

[CR12] Bonhoeffer T, Grinvald A (1993). The layout of iso-orientation domains in area 18 of cat visual cortex: optical imaging reveals a pinwheel-like organization. J Neurosci.

[CR13] Bonhoeffer T, Grinvald A, Toga AW, Mazziotta JC (1996). Optical imaging based on intrinsic signals. The methodology. Brain mapping: the methods.

[CR14] Bonhoeffer T, Kim DS, Malonek D, Shoham D, Grinvald A (1995). Optical imaging of the layout of functional domains in area 17 and across the area 17/18 border in cat visual cortex. Eur J Neurosci.

[CR15] Bortone DS, Olsen SR, Scanziani M (2014). Translaminar inhibitory cells recruited by layer 6 corticothalamic neurons suppress visual cortex. Neuron.

[CR16] Bosking WH, Zhang Y, Schofield B, Fitzpatrick D (1997). Orientation selectivity and the arrangement of horizontal connections in tree shrew striate cortex. J Neurosci.

[CR17] Boyd JD, Matsubara JA (1996). Laminar and columnar patterns of geniculocortical projections in the cat: relationship to cytochrome oxidase. J Comp Neurol.

[CR18] Braak H, Peters A, Jones EG (1984). Architecture as seen by Lipofuscin stains. Cerebral cortex, vol cellular components of the cerebral corte.

[CR19] Briggs F (2010). Organizing principles of cortical layer 6. Front Neural Circ.

[CR20] Budd JM (2004). How much feedback from visual cortex to lateral geniculate nucleus in cat: a perspective. Vis Neurosci.

[CR21] Bullier J, Henry GH (1979). Laminar distribution of first-order neurons and afferent terminals in cat striate cortex. J Neurophysiol.

[CR22] Buzás P, Eysel UT, Kisvárday ZF (1998). Functional topography of single cortical cells: an intracellular approach combined with optical imaging. Brain Res Brain Res Protoc.

[CR23] Buzás P, Kovacs K, Ferecsko AS, Budd JM, Eysel UT, Kisvárday ZF (2006). Model-based analysis of excitatory lateral connections in the visual cortex. J Comp Neurol.

[CR24] Chisum HJ, Mooser F, Fitzpatrick D (2003). Emergent properties of layer 2/3 neurons reflect the collinear arrangement of horizontal connections in tree shrew visual cortex. J Neurosci.

[CR25] Conley M, Fitzpatrick D, Diamond IT (1984). The laminar organization of the lateral geniculate body and the striate cortex in the tree shrew (*Tupaia glis*). J Neurosci.

[CR26] Creutzfeldt O, Mucke L, Bang-Olsen R (1980). Responses of claustral neurons to visual stimulation. Exp Brain Res.

[CR27] Crick F, Koch C (2005). What is the function of the claustrum?. Philos Trans R Soc Lond B Biol Sci.

[CR28] Crook JM, Eysel UT (1992). GABA-induced inactivation of functionally characterized sites in cat visual cortex (area 18): effects on orientation tuning. J Neurosci.

[CR29] Crook JM, Eysel UT, Machemer HF (1991). Influence of GABA-induced remote inactivation on the orientation tuning of cells in area 18 of feline visual cortex: a comparison with area 17. Neuroscience.

[CR30] Cudeiro J, Sillito AM (2006). Looking back: corticothalamic feedback and early visual processing. Trends Neurosci.

[CR31] da Costa NM, Martin KA (2009). Selective targeting of the dendrites of corticothalamic cells by thalamic afferents in area 17 of the cat. J Neurosci.

[CR32] da Costa NM, Fursinger D, Martin KA (2010). The synaptic organization of the claustral projection to the cat’s visual cortex. J Neurosci.

[CR33] Fitzpatrick D, Itoh K, Diamond IT (1983). The laminar organization of the lateral geniculate body and the striate cortex in the squirrel monkey (*Saimiri sciureus*). J Neurosci.

[CR34] Gabbott PL, Martin KA, Whitteridge D (1987). Connections between pyramidal neurons in layer 5 of cat visual cortex (area 17). J Comp Neurol.

[CR35] Garey LJ (1965). Interrelationships of the visual cortex and superior colliculus in the cat. Nature.

[CR36] Gilbert CD (1977). Laminar differences in receptive field properties of cells in cat primary visual cortex. J Physiol.

[CR37] Gilbert CD, Kelly JP (1975). The projections of cells in different layers of the cat’s visual cortex. J Comp Neurol.

[CR38] Gilbert CD, Wiesel TN (1979). Morphology and intracortical projections of functionally characterised neurones in the cat visual cortex. Nature.

[CR39] Gilbert CD, Wiesel TN (1983). Clustered intrinsic connections in cat visual cortex. J Neurosci.

[CR40] Gilbert CD, Wiesel TN (1989). Columnar specificity of intrinsic horizontal and corticocortical connections in cat visual cortex. J Neurosci.

[CR41] Giurcăneanu CD, Tăbuş I (2004). Cluster structure inference based on clustering stability with applications to microarray data analysis. EURASIP J Adv Signal Process.

[CR42] Grieve KL, Sillito AM (1991). The length summation properties of layer VI cells in the visual cortex and hypercomplex cell end zone inhibition. Exp Brain Res.

[CR43] Grieve KL, Sillito AM (1991). A re-appraisal of the role of layer VI of the visual cortex in the generation of cortical end inhibition. Exp Brain Res.

[CR44] Grieve KL, Sillito AM (1995). Differential properties of cells in the feline primary visual cortex providing the corticofugal feedback to the lateral geniculate nucleus and visual claustrum. J Neurosci.

[CR45] Gusfield D (2002). Partition-distance: a problem and class of perfect graphs arising in clustering. Inf Proces Lett.

[CR46] Harvey AR (1980). The afferent connexions and laminar distribution of cells in area 18 of the cat. J Physiol.

[CR47] Harvey AR (1980). A physiological analysis of subcortical and commissural projections of areas 17 and 18 of the cat. J Physiol.

[CR48] Henry GH, Harvey AR, Lund JS (1979). The afferent connections and laminar distribution of cells in the cat striate cortex. J Comp Neurol.

[CR49] Hirsch JA, Gallagher CA, Alonso JM, Martinez LM (1998). Ascending projections of simple and complex cells in layer 6 of the cat striate cortex. J Neurosci.

[CR50] Hubel DH, Wiesel TN (1962). Receptive fields, binocular interaction and functional architecture in the cat’s visual cortex. J Physiol.

[CR51] Hubel DH, Wiesel TN (1972). Laminar and columnar distribution of geniculo-cortical fibers in the macaque monkey. J Comp Neurol.

[CR52] Hübener M, Schwarz C, Bolz J (1990). Morphological types of projection neurons in layer 5 of cat visual cortex. J Comp Neurol.

[CR53] Ichinohe N, Matsushita A, Ohta K, Rockland KS (2010). Pathway-specific utilization of synaptic zinc in the macaque ventral visual cortical areas. Cereb Cortex.

[CR54] Jones EG (2007). The thalamus.

[CR55] Kaneko T, Fujiyama F, Hioki H (2002). Immunohistochemical localization of candidates for vesicular glutamate transporters in the rat brain. J Comp Neurol.

[CR56] Karube F, Kisvárday ZF (2011). Axon topography of layer IV spiny cells to orientation map in the cat primary visual cortex (area 18). Cereb Cortex.

[CR57] Kasper EM, Larkman AU, Lübke J, Blakemore C (1994). Pyramidal neurons in layer 5 of the rat visual cortex. I. Correlation among cell morphology, intrinsic electrophysiological properties, and axon targets. J Comp Neurol.

[CR58] Katz LC (1987). Local circuitry of identified projection neurons in cat visual cortex brain slices. J Neurosci.

[CR59] Khibnik LA, Tritsch NX, Sabatini BL (2014). A direct projection from mouse primary visual cortex to dorsomedial striatum. PLoS One.

[CR60] Kisvárday ZF, Eysel UT (1992). Cellular organization of reciprocal patchy networks in layer III of cat visual cortex (area 17). Neuroscience.

[CR61] Kisvárday ZF, Kim DS, Eysel UT, Bonhoeffer T (1994). Relationship between lateral inhibitory connections and the topography of the orientation map in cat visual cortex. Eur J Neurosci.

[CR62] Kisvárday ZF, Toth E, Rausch M, Eysel UT (1997). Orientation-specific relationship between populations of excitatory and inhibitory lateral connections in the visual cortex of the cat. Cereb Cortex.

[CR63] Kumar P, Ohana O (2008). Inter- and intralaminar subcircuits of excitatory and inhibitory neurons in layer 6a of the rat barrel cortex. J Neurophysiol.

[CR64] LeVay S, Gilbert CD (1976). Laminar patterns of geniculocortical projection in the cat. Brain Res.

[CR65] LeVay S, Sherk H (1981). The visual claustrum of the cat. I. Structure and connections. J Neurosci.

[CR66] Lorente de No R (1992). The cerebral cortex of the mouse (a first contribution—the “acoustic” cortex). Somatosens Mot Res.

[CR67] Lund JS, Boothe RG (1975). Interlaminar connections and pyramidal neuron organisation in the visual cortex, area 17, of the Macaque monkey. J Comp Neurol.

[CR68] Lund JS, Henry GH, MacQueen CL, Harvey AR (1979). Anatomical organization of the primary visual cortex (area 17) of the cat. A comparison with area 17 of the macaque monkey. J Comp Neurol.

[CR69] Malach R, Amir Y, Harel M, Grinvald A (1993). Relationship between intrinsic connections and functional architecture revealed by optical imaging and in vivo targeted biocytin injections in primate striate cortex. Proc Natl Acad Sci USA.

[CR70] Martin KA, Whitteridge D (1984). Form, function and intracortical projections of spiny neurones in the striate visual cortex of the cat. J Physiol.

[CR71] Martin KA, Roth S, Rusch ES (2014). Superficial layer pyramidal cells communicate heterogeneously between multiple functional domains of cat primary visual cortex. Nat Commun.

[CR72] Masamizu Y, Tanaka YR, Tanaka YH, Hira R, Ohkubo F, Kitamura K, Isomura Y, Okada T, Matsuzaki M (2014). Two distinct layer-specific dynamics of cortical ensembles during learning of a motor task. Nat Neurosci.

[CR73] McCourt ME, Boyapati J, Henry GH (1986). Layering in lamina 6 of cat striate cortex. Brain Res.

[CR74] McGuire BA, Hornung JP, Gilbert CD, Wiesel TN (1984). Patterns of synaptic input to layer 4 of cat striate cortex. J Neurosci.

[CR75] Mendizabal-Zubiaga JL, Reblet C, Bueno-Lopez JL (2007). The underside of the cerebral cortex: layer V/VI spiny inverted neurons. J Anat.

[CR76] Mercer A, West DC, Morris OT, Kirchhecker S, Kerkhoff JE, Thomson AM (2005). Excitatory connections made by presynaptic cortico-cortical pyramidal cells in layer 6 of the neocortex. Cereb Cortex.

[CR77] Movshon JA, Thompson ID, Tolhurst DJ (1978). Spatial and temporal contrast sensitivity of neurones in areas 17 and 18 of the cat’s visual cortex. J Physiol.

[CR78] Muir DR, Da Costa NM, Girardin CC, Naaman S, Omer DB, Ruesch E, Grinvald A, Douglas RJ (2011). Embedding of cortical representations by the superficial patch system. Cereb Cortex.

[CR79] Murphy PC, Sillito AM (1986). Continuity of orientation columns between superficial and deep laminae of the cat primary visual cortex. J Physiol.

[CR80] Murphy PC, Sillito AM (1996). Functional morphology of the feedback pathway from area 17 of the cat visual cortex to the lateral geniculate nucleus. J Neurosci.

[CR81] Murphy PC, Duckett SG, Sillito AM (2000). Comparison of the laminar distribution of input from areas 17 and 18 of the visual cortex to the lateral geniculate nucleus of the cat. J Neurosci.

[CR82] Nahmani M, Erisir A (2005). VGluT2 immunochemistry identifies thalamocortical terminals in layer 4 of adult and developing visual cortex. J Comp Neurol.

[CR83] Ohki K, Matsuda Y, Ajima A, Kim DS, Tanaka S (2000). Arrangement of orientation pinwheel centers around area 17/18 transition zone in cat visual cortex. Cereb Cortex.

[CR84] O’Leary JL (1941). Structure of the area striata of the cat. J Comp Neurol.

[CR85] Olsen SR, Bortone DS, Adesnik H, Scanziani M (2012). Gain control by layer six in cortical circuits of vision. Nature.

[CR86] Orban GA (1984). Neuronal operations in the visual cortex. vol 11. Studies of brain function.

[CR87] Otsuka R, Hassler R (1962). On the structure and segmentation of the cortical center of vision in the cat. Archiv fur Psychiatrie und Nervenkrankheiten, vereinigt mit Zeitschrift fur die gesamte Neurologie und Psychiatrie.

[CR88] Payne BR, Peters A, Payne BR, Peters A (2002). The concept of cat primary visual cortex. The cat primary visual cortex.

[CR89] Pichon F, Nikonenko I, Kraftsik R, Welker E (2012). Intracortical connectivity of layer VI pyramidal neurons in the somatosensory cortex of normal and barrelless mice. Eur J Neurosci.

[CR90] Ringach DL, Hawken MJ, Shapley R (1997). Dynamics of orientation tuning in macaque primary visual cortex. Nature.

[CR91] Rockland KS (2014). Zinc-positive and zinc-negative connections of the claustrum. Front Syst Neurosci.

[CR92] Rockland KS, Lund JS (1982). Widespread periodic intrinsic connections in the tree shrew visual cortex. Science.

[CR93] Rockland KS, Lund JS (1983). Intrinsic laminar lattice connections in primate visual cortex. J Comp Neurol.

[CR94] Schmidt KE, Goebel R, Lowel S, Singer W (1997). The perceptual grouping criterion of colinearity is reflected by anisotropies of connections in the primary visual cortex. Eur J Neurosci.

[CR95] Sherk H, LeVay S (1981). Visual claustrum: topography and receptive field properties in the cat. Science.

[CR96] Shmuel A, Grinvald A (2000). Coexistence of linear zones and pinwheels within orientation maps in cat visual cortex. Proc Natl Acad Sci USA.

[CR97] Sillito AM, Cudeiro J, Jones HE (2006). Always returning: feedback and sensory processing in visual cortex and thalamus. Trends Neurosci.

[CR98] Sincich LC, Blasdel GG (2001). Oriented axon projections in primary visual cortex of the monkey. J Neurosci.

[CR99] Smythies J (2015). On the function of object cells in the claustrum-key components in information processing in the visual system?. Front Cell Neurosci.

[CR100] Somogyi P, Lam DKT, Gilbert CD (1989). synaptic organization of GABAergic neurons and GABA receptors in the lateral geniculate nucleus and visual cortex. neural mechanisms of visual perception.

[CR101] Somogyi P, Freund TF, Heimer L, Zaborszky L (1989). Immunocytochemistry and synaptic relationship of physiologically characterized HRP-filled neurons. Neuroanatomical tract tracing methods.

[CR102] Staiger JF, Zilles K, Freund TF (1996). Recurrent axon collaterals of corticothalamic projection neurons in rat primary somatosensory cortex contribute to excitatory and inhibitory feedback-loops. Anat Embryol (Berl).

[CR103] Stratford KJ, Tarczy-Hornoch K, Martin KAC, Bannister NJ, Jack JJ (1996). Excitatory synaptic inputs to spiny stellate cells in cat visual cortex. Nature.

[CR104] Swindale NV, Matsubara JA, Cynader MS (1987). Surface organization of orientation and direction selectivity in cat area 18. J Neurosci.

[CR105] Tanaka YR, Tanaka YH, Konno M, Fujiyama F, Sonomura T, Okamoto-Furuta K, Kameda H, Hioki H, Furuta T, Nakamura KC, Kaneko T (2011). Local connections of excitatory neurons to corticothalamic neurons in the rat barrel cortex. J Neurosci.

[CR106] Tarczy-Hornoch K, Martin KA, Stratford KJ, Jack JJ (1999). Intracortical excitation of spiny neurons in layer 4 of cat striate cortex in vitro. Cereb Cortex.

[CR107] Thomson AM (2010). Neocortical layer 6, a review. Front Neuroanat.

[CR108] Tian P, Devor A, Sakadzic S, Dale AM, Boas DA (2011). Monte Carlo simulation of the spatial resolution and depth sensitivity of two-dimensional optical imaging of the brain. J Biomed Opt.

[CR109] Tsumoto T, Suda K (1980). Three groups of cortico-geniculate neurons and their distribution in binocular and monocular segments of cat striate cortex. J Comp Neurol.

[CR110] Tsumoto T, Creutzfeldt OD, Legendy CR (1978). Functional organization of the corticofugal system from visual cortex to lateral geniculate nucleus in the cat (with an appendix on geniculo-cortical mono-synaptic connections). Exp Brain Res.

[CR111] Tusa RJ, Rosenquist AC, Palmer LA (1979). Retinotopic organization of areas 18 and 19 in the cat. J Comp Neurol.

[CR112] Usrey WM, Fitzpatrick D (1996). Specificity in the axonal connections of layer VI neurons in tree shrew striate cortex: evidence for distinct granular and supragranular systems. J Neurosci.

[CR113] Velez-Fort M, Rousseau CV, Niedworok CJ, Wickersham IR, Rancz EA, Brown AP, Strom M, Margrie TW (2014). The stimulus selectivity and connectivity of layer six principal cells reveals cortical microcircuits underlying visual processing. Neuron.

[CR114] Ward JH (1963). Hierarchical grouping to optimize an objective function. J Am Stat Assoc.

[CR115] Watakabe A, Hirokawa J, Ichinohe N, Ohsawa S, Kaneko T, Rockland KS, Yamamori T (2012). Area-specific substratification of deep layer neurons in the rat cortex. J Comp Neurol.

[CR116] West DC, Mercer A, Kirchhecker S, Morris OT, Thomson AM (2006). Layer 6 cortico-thalamic pyramidal cells preferentially innervate interneurons and generate facilitating EPSPs. Cereb Cortex.

[CR117] Wiser AK, Callaway EM (1996). Contributions of individual layer 6 pyramidal neurons to local circuitry in macaque primary visual cortex. J Neurosci.

[CR118] Yoshioka T, Blasdel GG, Levitt JB, Lund JS (1996). Relation between patterns of intrinsic lateral connectivity, ocular dominance, and cytochrome oxidase-reactive regions in macaque monkey striate cortex. Cereb Cortex.

[CR119] Yousef T, Bonhoeffer T, Kim DS, Eysel UT, Toth E, Kisvárday ZF (1999). Orientation topography of layer 4 lateral networks revealed by optical imaging in cat visual cortex (area 18). Eur J Neurosci.

[CR120] Zarrinpar A, Callaway EM (2006). Local connections to specific types of layer 6 neurons in the rat visual cortex. J Neurophysiol.

[CR121] Zhang ZW, Deschenes M (1997). Intracortical axonal projections of lamina VI cells of the primary somatosensory cortex in the rat: a single-cell labeling study. J Neurosci.

