# Supplementary material for: Axon topography of layer 6 spiny cells to orientation map in the primary visual cortex of the cat (area 18)
Source: Brain Struct Funct. 2016 Aug 18;222(3):1401–26. doi: 10.1007/s00429-016-1284-z (PMC5368233; doi:10.1007/s00429-016-1284-z)
Supplement: Supplementary file 1 — Supplementary material 1 (DOCX 28 kb) [file 429_2016_1284_MOESM1_ESM.docx]

**Supplementary figure legends**

Axon topography of layer 6 spiny cells to orientation map in the primary visual cortex of the cat (area 18). Fuyuki Karube, Katalin Sári, Zoltán F. Kisvárday

**Supplementary figure 1**

(a), Superposition of surface image of the cortex and section contours (white lines) taken from the same area. Red circles show location of unit recordings, blue circles represent reference penetrations used for alignment between optical images and cell reconstructions. Top and bottom edge of the image corresponds, respectively, to the medial margin of the hemisphere and lateral sulcus. Narrow separation between successive section contours indicates where the cortical surface is not flat. (b), orientation preference map of the same area shown in A. Note that close to the medial margin and the lateral sulcus the orientation map contains distortion, hence labelled neurons in these areas were excluded from analysis.

**Supplementary figure 2**

Locations of reconstructed layer 6 neurons. Twenty-three layer 6 neurons were reconstructed from 6 cats. The cell body location of each reconstructed cell is marked by triangle. Note that almost all cells are located in the middle zone of the sections which represents the least convoluted part of the exposed cortex. However, a few axon (in blue) was found close to the marginal zone where functional maps are difficult to interpret (see also main text and Supplementary Fig. 1). If >30% of boutons of the reconstructed cell overlapped with the marginal zone, that cell was omitted from the analysis of orientation preference. According to this criteria cells #12062 and #1503 (in blue) were excluded from the orientation analysis while cell #9042 which provided <30% of boutons (in blue) outside the flat area was included in the orientation analysis. Gray lines indicate section contours.

**Supplementary figure 3**

(a), Sholl analysis of basal dendrite distribution. No clear difference between cell types was observed as shown by the strongly overlapping graphs. (b), Sholl analysis of apical dendrite distribution. As is expected, the plots support apical dendrite types defined by the cluster analysis.

**Supplementary figure 4**

Horizontal (a) and vertical (b) distribution of boutons to L6 cell Types. (a1), Proportion of boutons plotted against horizontal distance from the parent soma (mean and SD are represented by lines and shaded areas, respectively). Most boutons of Type Aa (black) and Type Ab (blue) were located proximal to the parent soma, whereas boutons of Type B (red) distributed rather evenly beyond 1 mm form soma location. Significant difference among types was observed at 300 – 450 μm and 850 – 1000 μm ranges. Difference between corresponding data points among types was examined using pairwise Wilcoxon test with Holm’s correction. (a2), Cumulative distribution of boutons differed significantly among the types (p<0.01 by Kolmogorov-Smirnov test with Bonferroni correction), Type B showed the smallest slope curve Reflecting longer horizontal bouton distribution. (b), Vertical distribution of boutons with respect to L3/4 lamina border. Vertical distance from the parent soma to each bouton was normalized with the mean distance between parent somata and the L3/4 border. Zero on the abscissa represents cell body location, +1 marks the L3/4 border and negative values are towards the white matter. (b1), Significant difference was observed between Type Aa and B at the distance of 0.5, where a sharp peak is present for Type Aa. (b2), Cumulative bouton proportion of the data shown in (b1). The early steep curves of Type Ab and B reflect a larger proportion of deep layer boutons (around zero distance or soma position) compared with those of Type Aa (which increase at around 0.5 to 1.0 distance corresponding to L4). Note that only Type B shows an increase beyond the L3/4 border.

**Supplementary figure 5**

Spatial distribution of bouton clusters of L4 and L6 cells. (a), relationship among 3D-distance from the soma to clusters, cluster weight and cluster rank. In L4 cells, rank 1 clusters were more proximally located than rank 2 clusters, whereas in L6, distance to rank 1 and rank 2 clusters was not significantly different. (b), the same plots as shown in A, except for distance was calculated in 2-D. For L4 cells, distribution of the plot was basically the same as in the case of 3-D distance. For L6 cells, rank 1 clusters were now proximally located than rank 2 clusters, suggesting wide vertical distribution of L6 rank 1 clusters. (c), vertical distance from the soma to rank 1 and rank 2 clusters in L6 cells. Rank 1 bouton clusters of Type Aa cells were distributed distally toward pia matter, reflecting intensive innervation to L4. Contrary, those of Type Ab and Type B were relatively near to the soma. Rank 2 clusters were also distributed wide range of vertical distance.

**Supplementary figure 6**

(a), Comparison of preferred orientation revealed by optical imaging vs. unit recording from L6 (N=65). Black line represents unity line and red dotted line represents linear fit of the observed data (regression coefficient = 0.87). The two data sets were highly correlated (R^2^= 0.68). In order to represent linear relationship clearly, grey data points are converted by adding 180 degrees. (b), Frequency distribution of orientation difference between unit recordings and optical imaging (the same data set as in A). Red curve shows the Gaussian fit. Kolmogorov-Smirnov test confirmed that the distribution was centered on zero degree.

**Supplementary figure 7**

Strength of bouton convergence. (a), (c), the number of boutons in convergent pixels was related to spatial separation of the parent somata and difference of preferred orientation between the parent soma and boutons, in L6 cells (a) and L4 cells (c). There is a tendency of an increased orientation difference of converging boutons from that of the parent somata with larger soma separation. For a better visibility, bouton number is shown according to color code and also indicated by the size of circles. Strong convergence by large number of boutons was often observed for close cell pairs and iso-orientation domains in L6 cells as well as for distant cell pairs and non-iso-orientation domains. Since L4 cells provided intensive innervation toward cross-orientation domains as well as iso-orientation domains, strong convergence occurred more frequently. (b), (d), relationship between strength of convergence and distance from the parent soma to boutons in L6 (b) and L4 (d) cells.　For L6, most converging boutons had < 500 μm separation from the parent somata and an almost equal representation of orientation differences. For L4, an increased presence of converging boutons with large orientation differences was seen at >500 μm separation.

**Supplementary figure 8**

Distribution of vesicular glutamate transporter 2 (VGluT2) positive (thalamocortical) terminals in cat area 18. (a), Low magnification image of VGluT2 immunoreactivity. Layer boundary (line) was determined on the basis of adjacent Nissl stained section (not shown). VGluT2 positive terminals (brown particles) were found at high density in L4 and low to medium density in L6. (b), High magnification image of the framed area shown in (a). Individual immunopositive terminals are clearly distinguishable. At some places, VGluT2 positive terminals showed clustering (arrowhead). (c), Double immunohistochemistry to VGluT2 (black) and parvalbumin (PV, brown) revealed VGluT2 clusters correspond to the location of targeted PV positive cell bodies and proximal dendrites. (d), Density distribution of VGluT2 positive terminals in upper, middle, and lower third tiers of L6. Each tier contained a rather similar density of terminals, although the middle tier of L6 showed statistically higher values (p<0.05). Scale bar, 500 µm in (a), and 10 µm in (b) and (c).
