# Supplementary figures and images for: Axon topography of layer 6 spiny cells to orientation map in the primary visual cortex of the cat (area 18)
Source: Brain Struct Funct. 2016 Aug 18;222(3):1401–26. doi: 10.1007/s00429-016-1284-z (PMC5368233; doi:10.1007/s00429-016-1284-z)

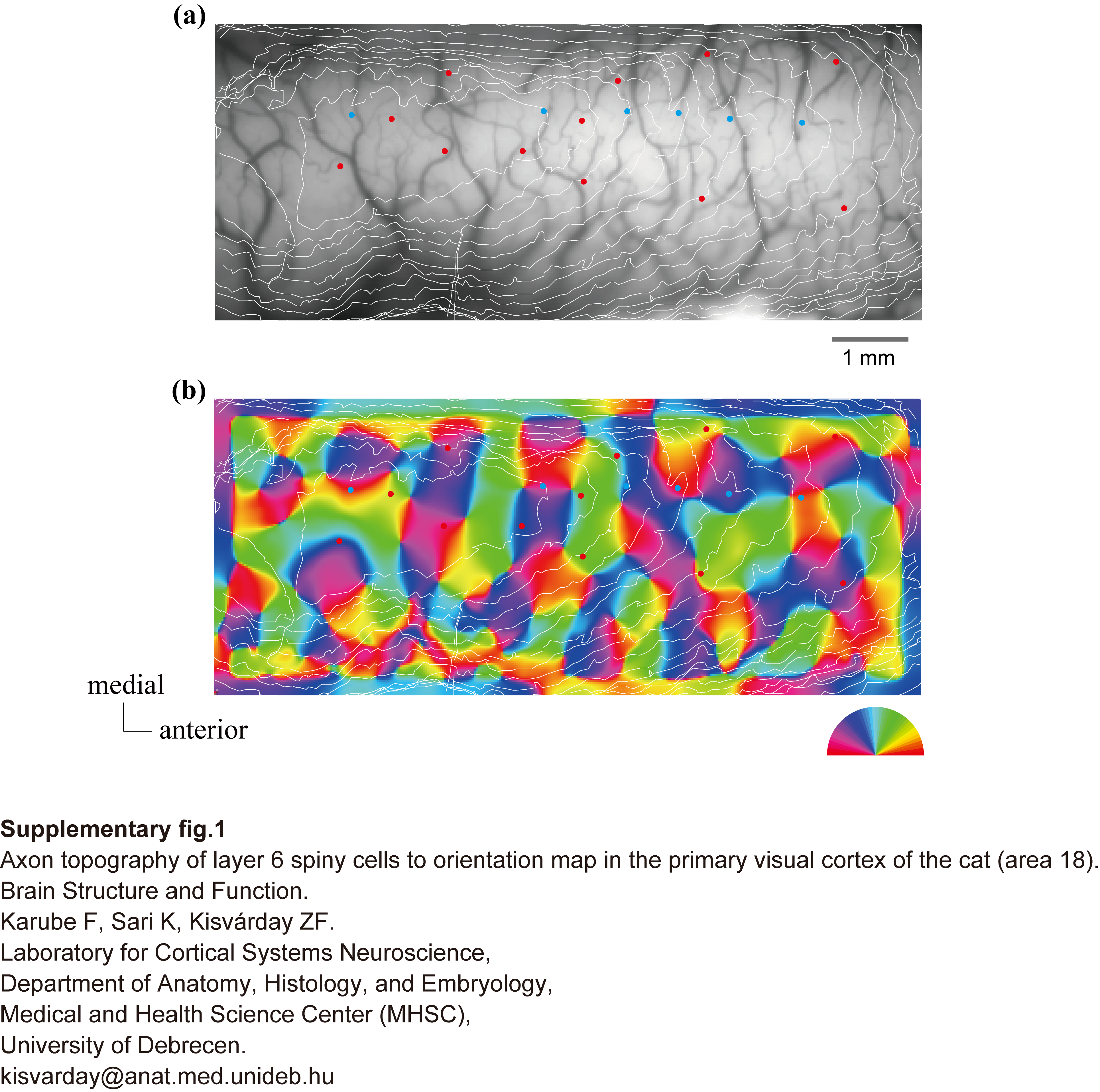

Supplement: Supplementary file 2 — Supplementary material 2 (TIFF 7533 kb) [file 429_2016_1284_MOESM2_ESM.tif]

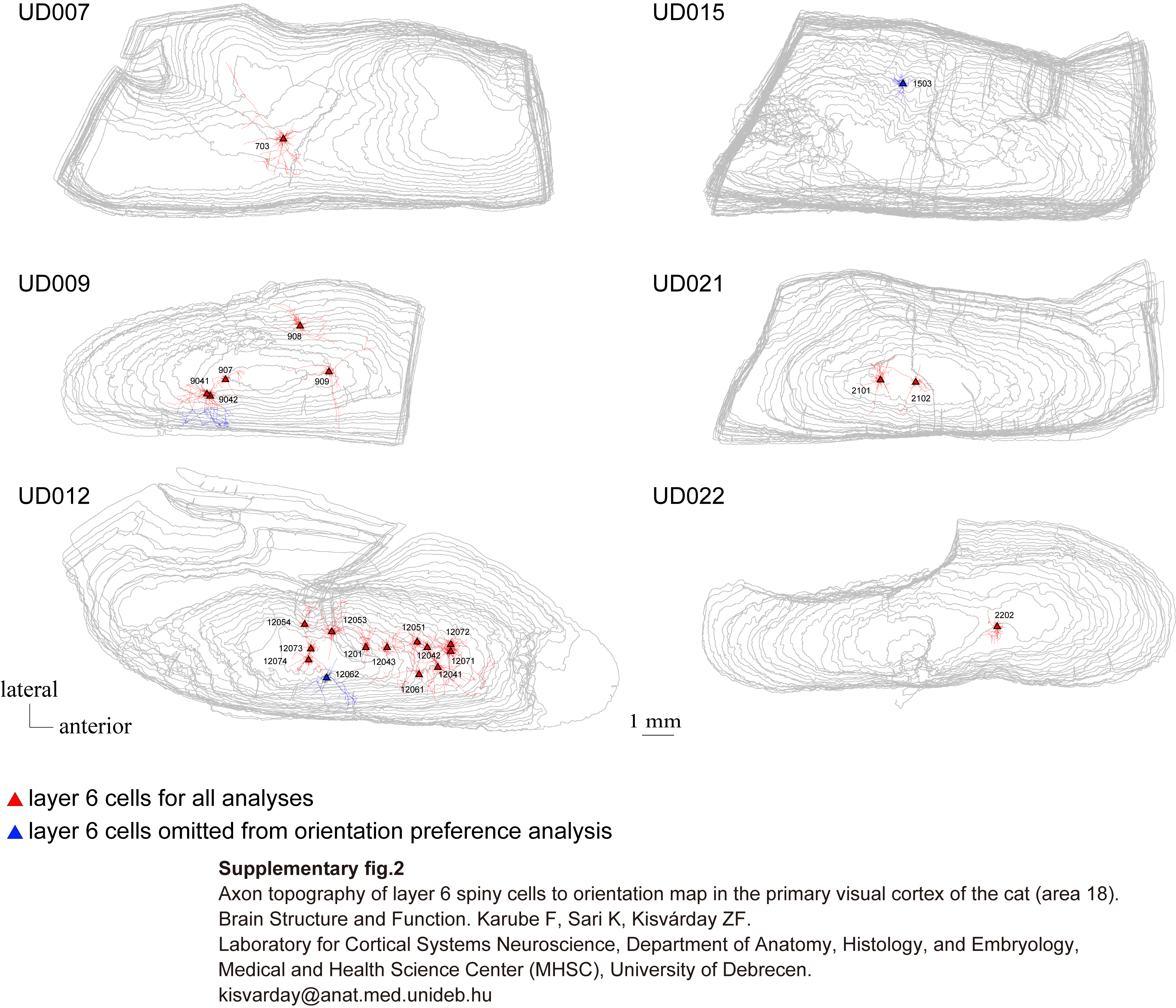

Supplement: Supplementary file 3 — Supplementary material 3 (TIFF 2173 kb) [file 429_2016_1284_MOESM3_ESM.tif]

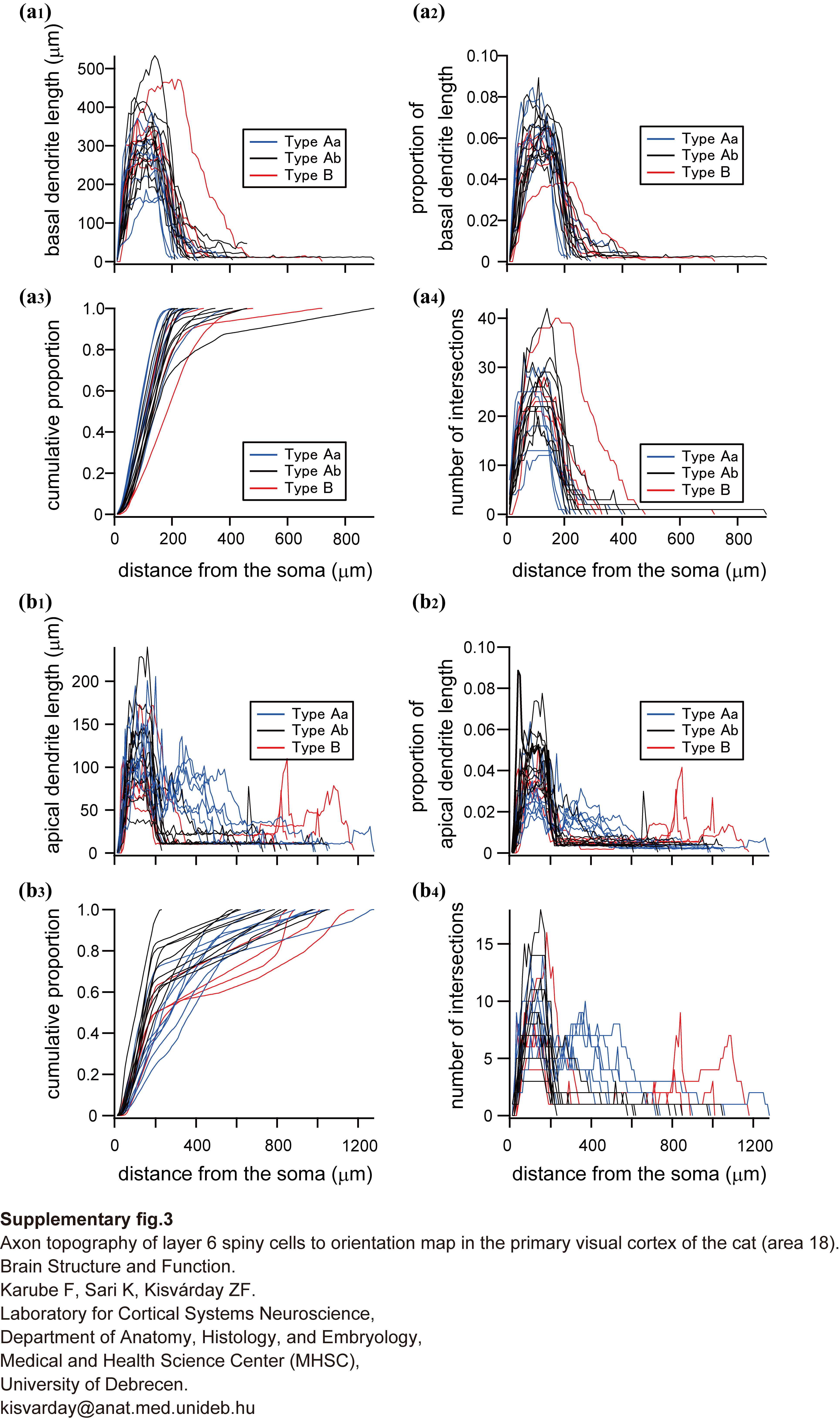

Supplement: Supplementary file 4 — Supplementary material 4 (TIFF 2541 kb) [file 429_2016_1284_MOESM4_ESM.tif]

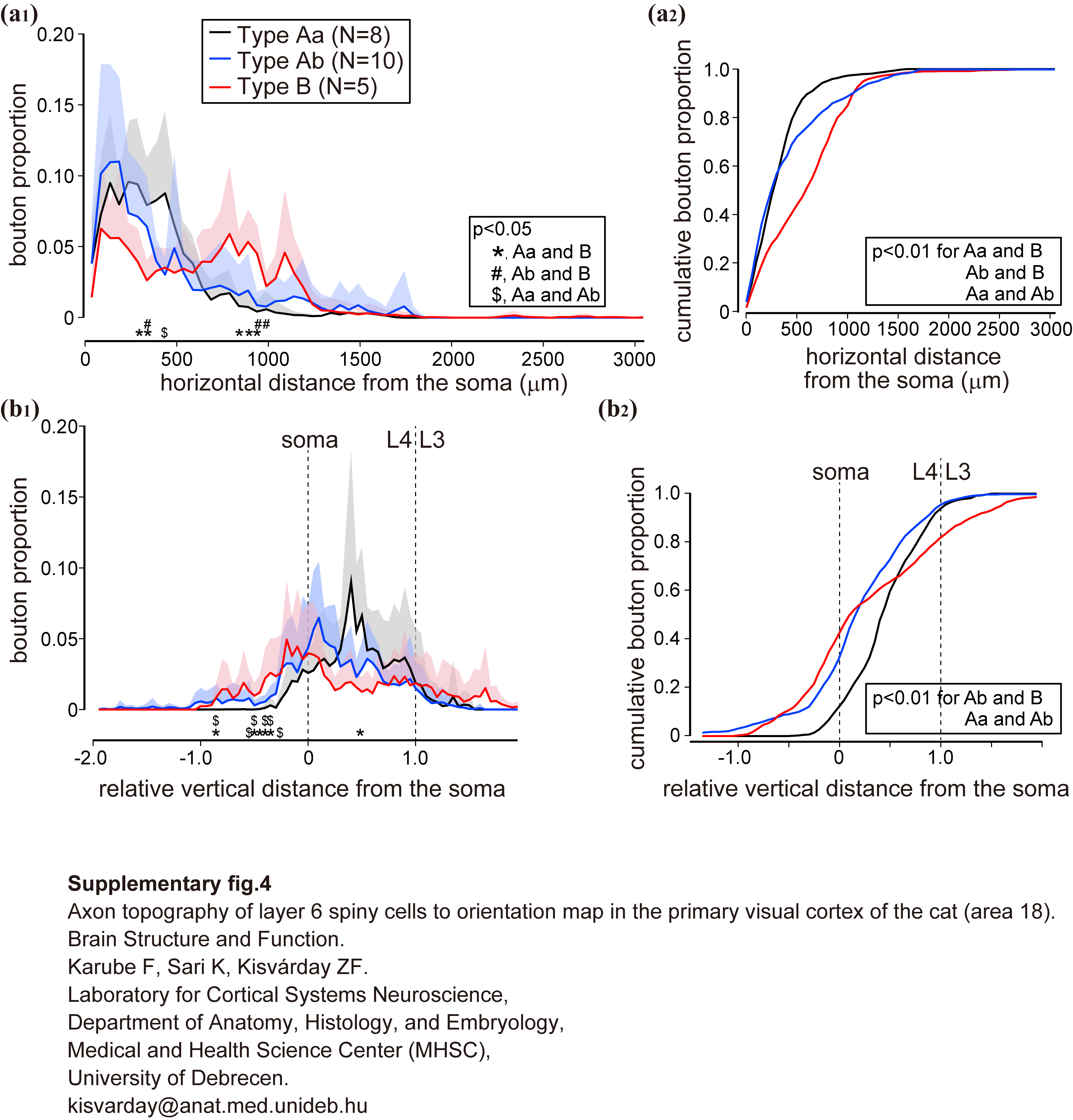

Supplement: Supplementary file 5 — Supplementary material 5 (TIFF 1756 kb) [file 429_2016_1284_MOESM5_ESM.tif]

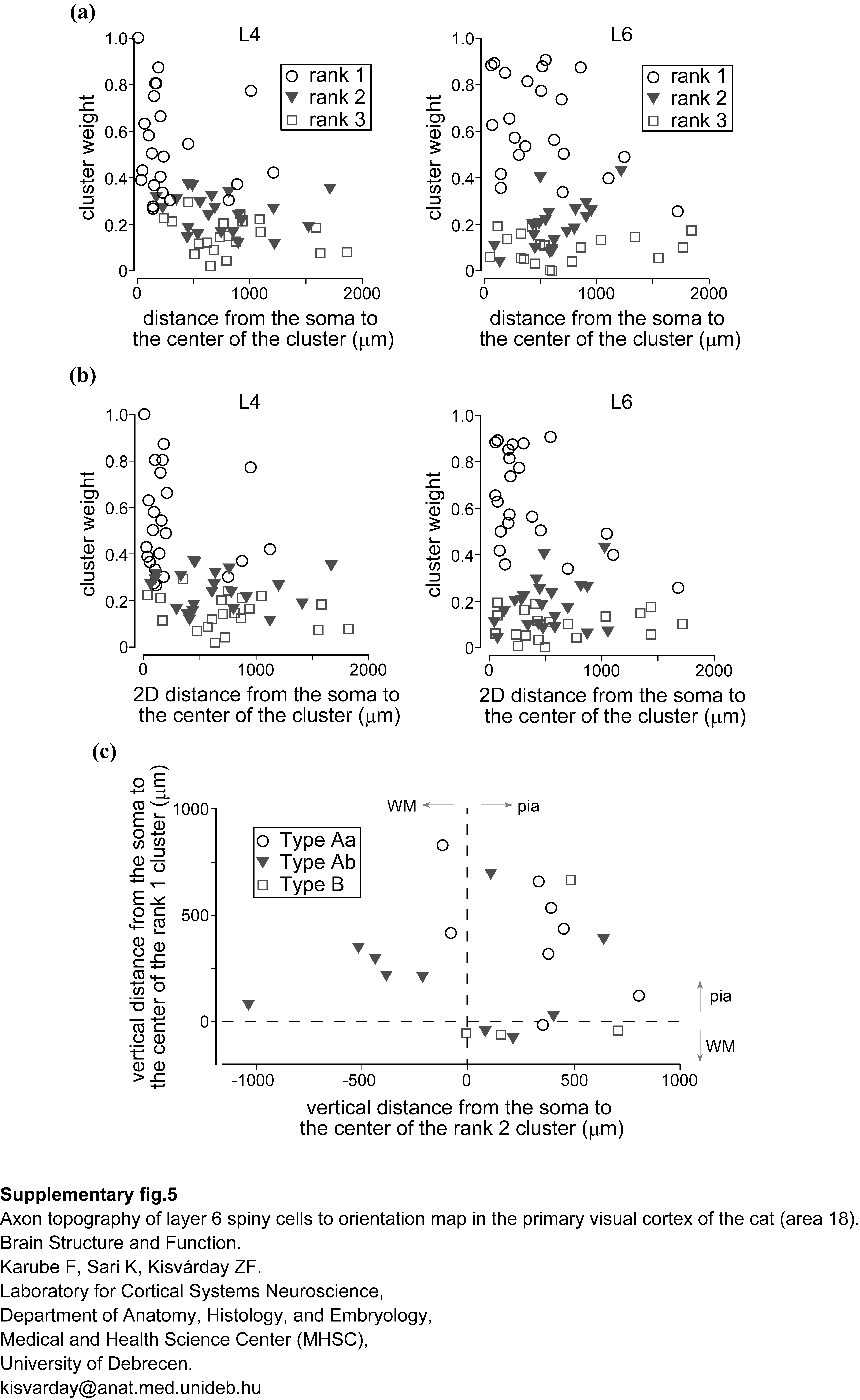

Supplement: Supplementary file 6 — Supplementary material 6 (TIFF 1814 kb) [file 429_2016_1284_MOESM6_ESM.tif]

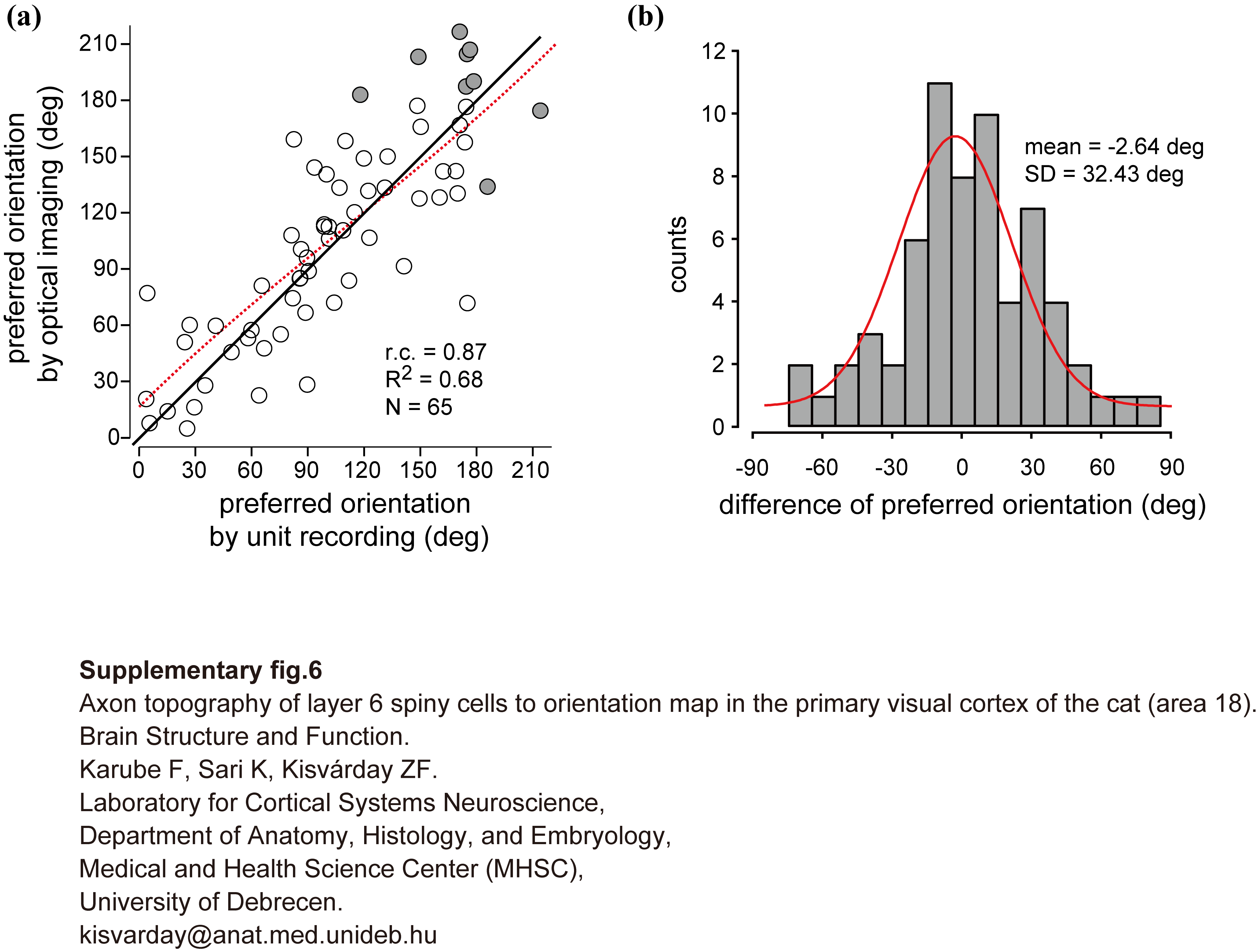

Supplement: Supplementary file 7 — Supplementary material 7 (TIFF 1104 kb) [file 429_2016_1284_MOESM7_ESM.tif]

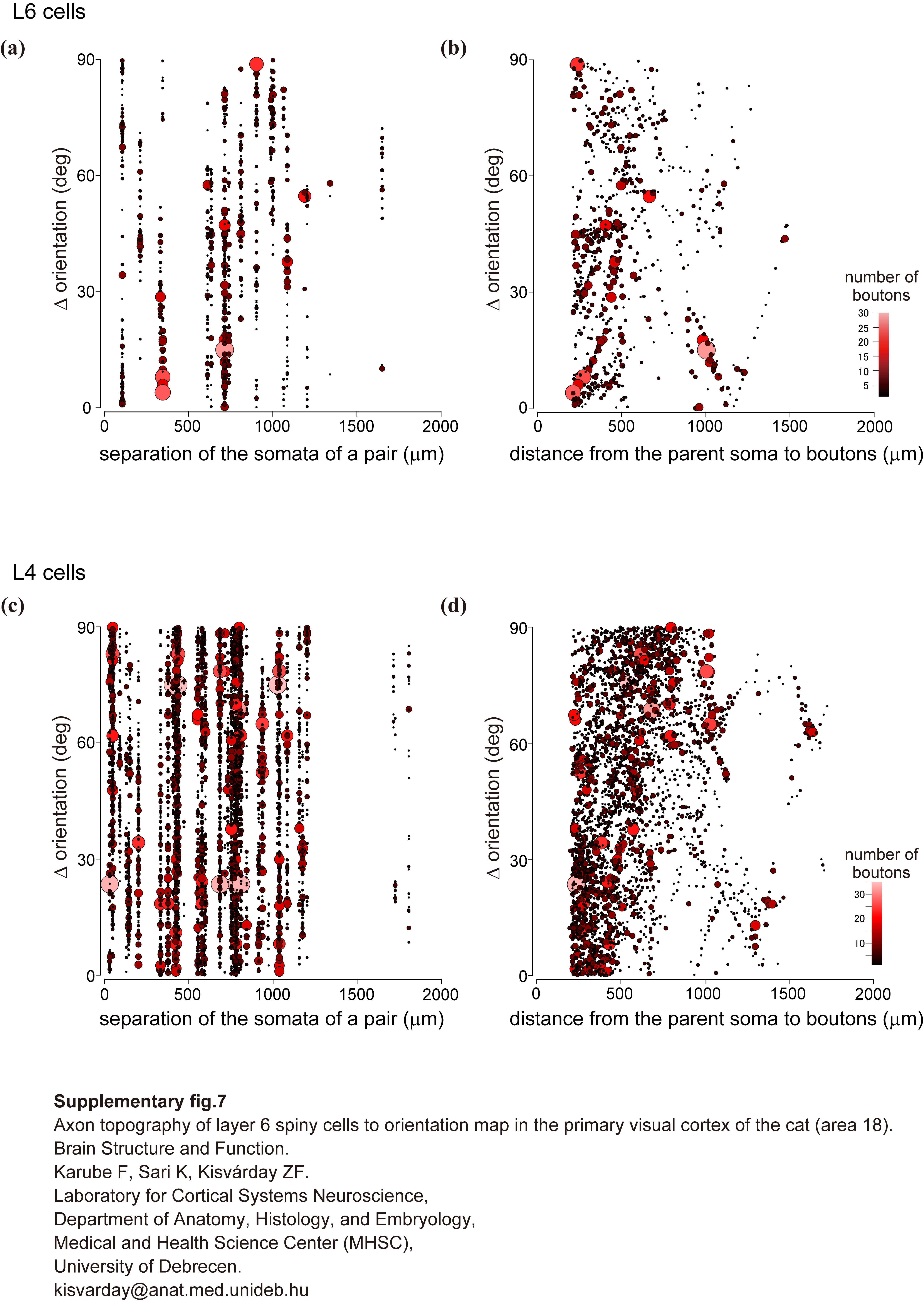

Supplement: Supplementary file 8 — Supplementary material 8 (TIFF 2652 kb) [file 429_2016_1284_MOESM8_ESM.tif]

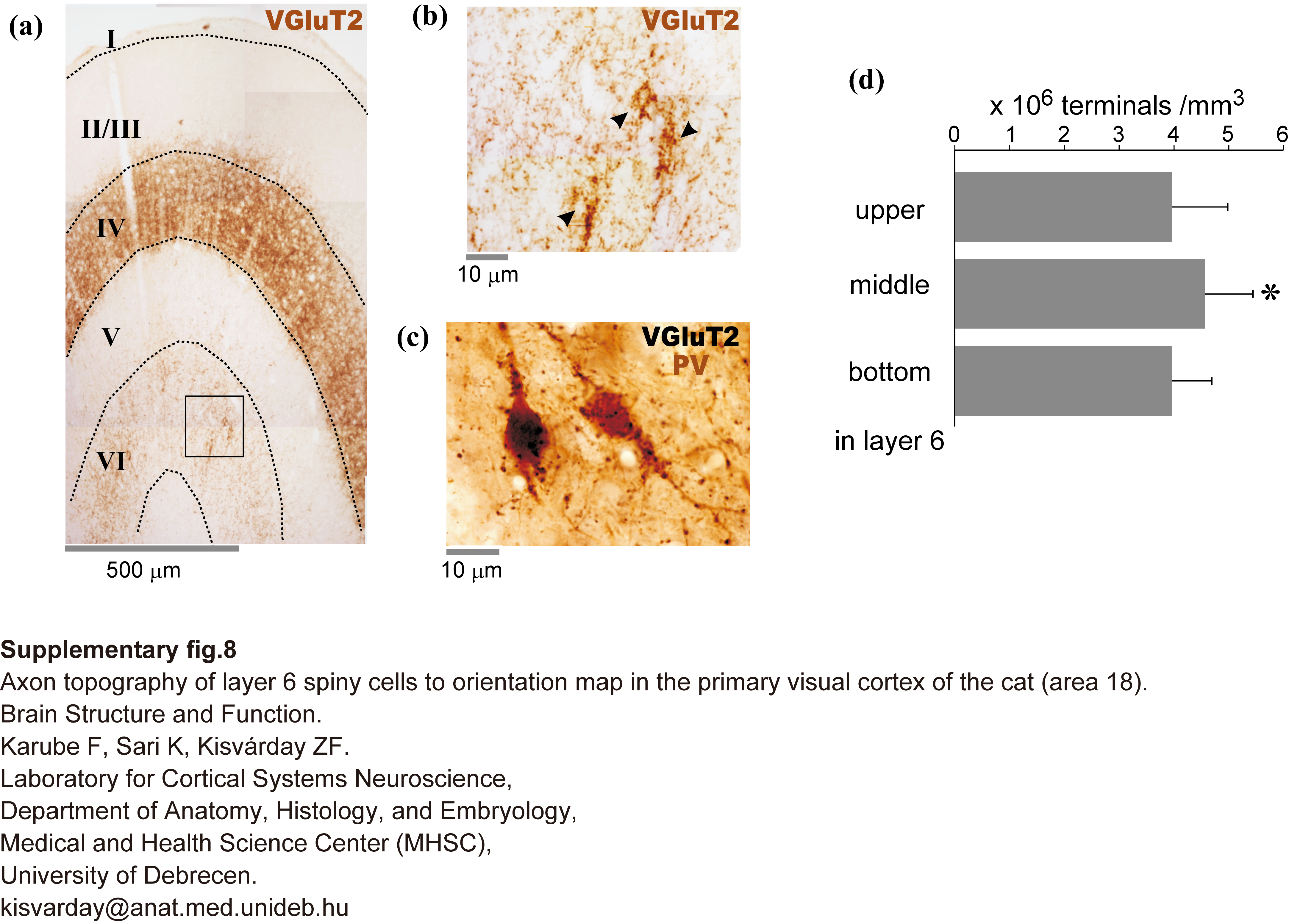

Supplement: Supplementary file 9 — Supplementary material 9 (TIFF 8914 kb) [file 429_2016_1284_MOESM9_ESM.tif]
